# The fructose survival hypothesis for obesity

**DOI:** 10.1098/rstb.2022.0230

**Published:** 2023-09-11

**Authors:** Richard J. Johnson, Miguel A. Lanaspa, L. Gabriela Sanchez-Lozada, Dean Tolan, Takahiko Nakagawa, Takuji Ishimoto, Ana Andres-Hernando, Bernardo Rodriguez-Iturbe, Peter Stenvinkel

**Affiliations:** ^1^ Department of Medicine, University of Colorado Anschutz Medical Center, Aurora, CO 80016, USA; ^2^ Department of Cardio-Renal Physiopathology, Instituto Nacional de Cardiología ‘Ignacio Chavez’, Mexico City 14080, Mexico; ^3^ Biology Department, Boston University, Boston, MA 02215, USA; ^4^ Department of Nephrology, Rakuwakai-Otowa Hospital, Kyoto 607-8062, Japan; ^5^ Department of Nephrology and Rheumatology, Aichi Medical University, Aichi 480-1103, Japan; ^6^ Department of Nephrology and Mineral Metabolism, Instituto Nacional de Ciencias Médicas y Nutrición ‘Salvador Zubirán’, Mexico City 14080, Mexico; ^7^ Department of Renal Medicine, Karolinska Institutet, Stockholm 171 77, Sweden

**Keywords:** obesity, fructose, metabolic syndrome, sugar, diabetes, uric acid

## Abstract

The fructose survival hypothesis proposes that obesity and metabolic disorders may have developed from over-stimulation of an evolutionary-based biologic response (survival switch) that aims to protect animals *in advance* of crisis. The response is characterized by hunger, thirst, foraging, weight gain, fat accumulation, insulin resistance, systemic inflammation and increased blood pressure. The process is initiated by the ingestion of fructose or by stimulating endogenous fructose production via the polyol pathway. Unlike other nutrients, fructose reduces the active energy (adenosine triphosphate) in the cell, while blocking its regeneration from fat stores. This is mediated by intracellular uric acid, mitochondrial oxidative stress, the inhibition of AMP kinase and stimulation of vasopressin. Mitochondrial oxidative phosphorylation is suppressed, and glycolysis stimulated. While this response is aimed to be modest and short-lived, the response in humans is exaggerated due to gain of ‘thrifty genes’ coupled with a western diet rich in foods that contain or generate fructose. We propose excessive fructose metabolism not only explains obesity but the epidemics of diabetes, hypertension, non-alcoholic fatty liver disease, obesity-associated cancers, vascular and Alzheimer's dementia, and even ageing. Moreover, the hypothesis unites current hypotheses on obesity. Reducing activation and/or blocking this pathway and stimulating mitochondrial regeneration may benefit health-span.

This article is part of a discussion meeting issue ‘Causes of obesity: theories, conjectures and evidence (Part I)’.

## 1. Introduction

An often overlooked approach to scientific discovery is to investigate how nature, and its accompanying powerhouse of evolution, have found solutions to vexing problems [1,2]. Such biomimetic approaches can provide insights for new therapies based on the ingenious approaches animals use in the wild to combat adversity [3,4]. However, evolution might also have an unwitting role in causing disease. Indeed, it has been suggested that genetic adaptations (thrifty genes) to assist survival in a world of scarce resources might ‘backfire’ in a world of plenty where it might increase the risk for obesity and diabetes [5].

Here, we discuss a recently discovered protective mechanism that we have named ‘the survival switch’ that is initiated *before* resources become scarce*.* Our work suggests that the effect is mediated by fructose, and that, unlike glucose whose primary biologic function is to provide an immediate fuel, that the primary function of fructose is to aid in the storage of fuel. These different biologic functions are consequences of how glucose and fructose metabolism modulate intracellular energy levels. We discuss the varied sources of fructose and how it mediates its biological actions. We also suggest that two events occurred that converted this protective pathway into one causing disease. The first was the acquisition of ‘thrifty genes’ (or more accurately the loss of genes that created a thrifty genotype), and the second event was the marked increase in foods that either contain or produce fructose. These two events have led to an overactivation of the ‘survival switch’ that we propose is driving both obesity and many of the ‘burden of life’ non-communicable diseases affecting us today.

## 2. Sources of fructose, the trigger of the survival switch

Fructose is a simple sugar that is the primary nutrient in fruit and honey. However, in the western diet, its main source is table sugar (sucrose), which consists of fructose and glucose bound together, and high fructose corn syrup (HFCS), which consists of a blended mixture of fructose and glucose, often with slightly higher concentrations of fructose as testing has suggested humans prefer slightly more fructose as it is sweeter than glucose. Today these ‘added sugars’ account for ≈15% of overall energy intake, with some groups ingesting as much as 20% or more [6].

**Figure 1.**
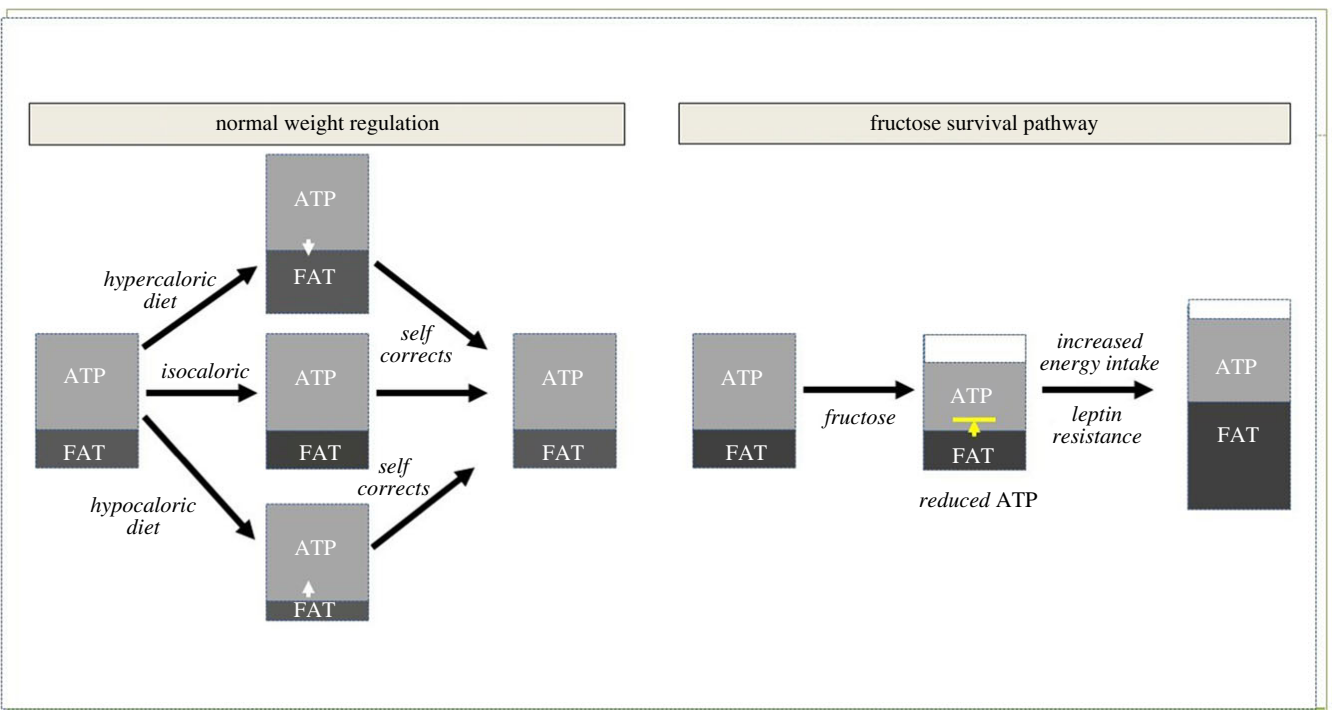
Fructose alters weight regulation. Under normal conditions, most animals tightly regulate their weight. If they are fed a hypercaloric diet they will gain weight, and if they are given a hypocaloric diet they will lose weight, but then they will spontaneously correct to their normal weight when they are allowed to resume their normal diet. By contrast, fructose acts by reducing intracellular ATP while at the same time blocking the replenishment of ATP from fat stores. Over a period of days to weeks, the animals develop leptin resistance, resulting in increased energy intake. However, ATP production stays low due to suppression from oxidative stress. As a consequence, the calories that are ingested are preferentially routed to generate fat. Over time ATP levels are repleted, but at the expense of a dramatic increase in fat stores. (Online version in colour.)

Fructose is also generated in the body from glucose (figure 1). This occurs when glucose levels (i.e. the substrate) are excessive, such as in diabetes, following the ingestion of high glycaemic carbohydrates, and by high carbohydrate diets [7–9]. The enzymatic conversion of glucose to fructose is called the polyol pathway and the rate-limiting enzyme is aldose reductase (AR). AR is activated by stress, such as dehydration (hyperosmolarity), starvation (such as with ischaemia) or with hypoxia. Fructose generation is also stimulated by salty foods and alcohol (both which raise serum osmolality) as well as by fructose itself [7,10,11]. Umami foods (rich in glutamate and nucleosides such as adenosine monophosphate (AMP) and inosine monophosphate (IMP)) also generate uric acid which stimulates fructose production [12,13]. All of these nutrients stimulate fructose generation in the liver where it is sufficient to fully activate the survival switch [14]. Fructose generation can also be stimulated in other organs, such as the brain, kidney, vasculature and heart. Indeed, fructose is generated locally in the brain with hyperglycaemia [9], in the heart with cardiac ischaemia [15], in the kidney with ischemic-reperfusion injury [16], in the circulation of the naked mole rat when it is crawling through hypoxic burrows [17], and in the placenta and fetus in the first trimester during the hypoxic period before placental circulation is fully intact [18]. Alcohol, due to its ability to raise osmolality, can also stimulate fructose production [11].

While most studies evaluating endogenous fructose production have been conducted in laboratory animals, there is data suggesting that endogenous fructose production in young lean adults may amount to greater than 5 g/day, with a tripling or more in fructose production rates following a high glycaemic soft drink [8]. Production is expected to be much higher in subjects on high glycaemic, high salt or on high sugar diets as it is known that AR is upregulated in this setting. High glycaemic and high salt diets can also induce a marked increased production of fructose in the brain [9,10]. Thus, fructose can be obtained and/or generated from the diet (sugar, HFCS, high glycaemic carbs, salty foods, umami foods, alcohol) as well as under conditions of stress (ischaemia, hypoxia and dehydration). Indeed, the three attractive tastes (sweet, salt, umami) all encourage intake of foods that generate fructose [7,10,12,19], while the bitter and sour tastes likely were developed to avoid foods that might carry toxins.

## 3. Fructose triggers the survival switch by lowering ATP levels

Weight is normally tightly regulated, especially for animals in the wild [20,21]. One of the strongest regulators appears to be lean (fat-free) body mass, and this measurement correlates with both energy intake and resting energy metabolism [22]. It is not surprising that muscle mass is a key characteristic the animal tries to protect, and in turn this is guided by the level of mitochondrial function [23]. As such, animals try to preserve intracellular ATP levels as this represents their active energy, and they do this by having metabolic flexibility in which any ATP that is expended can be rapidly replaced from ATP generated from nutrient intake or from fat stores (figure 1). In this setting, the administration of hypercaloric diet at levels that are not compensated acutely by an increase in energy expenditure will result in weight gain (and fat accumulation), while a hypocaloric diet will lead to weight loss (and a depletion of fat stores). In both conditions, energy balance is maintained and ATP levels preserved.

By contrast, fructose metabolism works differently, although it also follows the rules of energy balance. Specifically, fructose actively lowers intracellular ATP while at the same time reducing the ability to make new ATP (figure 1). Thus, the metabolic flexibility is blocked. ATP levels do not fall so far as to threaten survival, but drop enough to activate an alarm that usable energy stores are at risk of being depleted. For example, in studies in humans, ATP levels can fall 20 per cent in the liver following oral ingestion of fructose [24], and up to 60–70 per cent if given intravenously [25]. The level of ATP depletion relates to the concentration of fructose that the liver is exposed to, which relates to the amount ingested and the speed of absorption, which is greater when the fructose is given as a liquid [26,27]. This triggers a set of biological responses that result in increased energy intake. However, the continued suppression of mitochondrial function results in the calories being shunted to stored energy (fat). Eventually ATP levels are replaced, but at the expense of storing more fat such that overall energy levels (i.e. active and stored energy) are higher.

**Figure 2.**
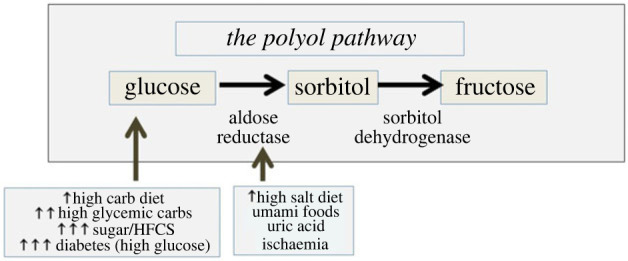
The endogenous production of fructose. Fructose is generated from glucose (its substrate) via the polyol pathway. Aldose reductase is the rate-limiting enzyme. The primary drivers for fructose production are high glucose levels, and factors that can stimulate aldose reductase activity. From a food perspective, this includes high glycaemic carbohydrates, salty foods, alcohol and umami foods such as beer. However, this pathway is also activated by stress, including ischaemia, hypoxia, dehydration and heat stress. (Online version in colour.)

The specific mechanism(s) by which fructose lowers intracellular ATP is shown in figure 2. Fructose is rapidly phosphorylated to fructose-1-phosphate by fructokinase C (also known as ketohexokinase-C, or KHK-C) that has no feedback system to protect ATP levels such that there is an acute fall in intracellular ATP and phosphate [28,29]. The fall in intracellular phosphate stimulates AMP deaminase-2 (AMPD2) that removes the AMP substrate [29], thereby slowing the regeneration of ATP, while at the same time stimulating the production of uric acid (from both the degradation of AMP to IMP as well as from de novo purine synthesis) [30,31]. This helps maintain a normal charge ratio of (AMP + ADP)/ATP. While some IMP could potentially be converted back to AMP and ATP by the purine salvage pathway [32], the production of uric acid appears to be favoured, and serum uric acid can increase by 0.3–2.0 mg dl^−1^ within the first hour following ingestion [30,33,34].

The production of uric acid by xanthine oxidoreductase generates oxidants (primarily hydrogen peroxide) [35] but uric acid itself also stimulates NADPH oxidase [36–40] that translocates to the mitochondria [41]. An increase in mitochondrial oxidative stress occurs from both NADPH oxidase and endogenous mitochondrial oxidative stress in conjunction with a simultaneous decrease in protective antioxidant systems (especially Nrf2) [41,42]. The oxidative stress reduces ATP production by inhibiting aconitase in the citric acid cycle [41,43–45] as well as blocking beta fatty acid oxidation by acetlating carnitine palmitoyl transferase-1a (CPT1*α* involved in fatty acid transport into mitochondria) [46] and enoyl Coa hydratase (an enzyme in the beta fatty acid oxidation pathway) [43,47], and blocks ATP regeneration by AMP-activated protein kinase [47,48]. The inhibition of aconitase stimulates the enzymes involved in lipogenesis [41,49] while acetate production by the microbiota in response to fructose is used to generate acetyl CoA that provides the substrate. Furthermore, there is some evidence that fructose metabolism can lead to the consumption of nicotinamide adenine dinucleotide (NAD+), reducing the NAD+/NADH ratio and affecting redox balance, and leading to a decrease in sirtuins, which may also confer metabolic effects [42,50] and augment the glycolytic response [51]. This may be responsible for the acetylation of CPT1*α* [46].

While mitochondrial oxidative phosphorylation and ATP generation is suppressed, the generation of fructose 1-phosphate stimulates the release of glucokinase from the nucleus, leading to glucose uptake and glycogen production, while the breakdown of fructose-1-phosphate generates glyceraldehyde and dihydroxyacetone phosphate. In a fasting state, this stimulates gluconeogenesis that can help provide glucose as an energy substrate [52], while in a fed state glycolysis is preferentially stimulated [53]. Lactate, for example, may account for as much of 25 per cent of the ingested fructose based on radiotracer studies [54]. While lactate can be used to generate acetyl CoA as a substrate for the citric acid cycle, when lactate builds up, it generates reactive oxygen species, impairs fatty acid uptake into the mitochondrial and reduces mitochondrial ATP generation [55]. The net stimulation of glycolysis by fructose [28,41,47,48] with suppression of oxygen consumption by the inhibition of oxidative phosphorylation likely was meant to provide survival benefits for animals at risk for hypoxia [17].

This trick of lowering intracellular ATP appears to be central to activating the survival response and disrupting weight regulation. In effect, the intake of calories is stimulated to correct for the ATP deficit, but the switch diverts the calories to fat. Eventually, ATP levels are repleted, but at the consequence of increasing adiposity. Over time there is a transition, as repeated oxidative stress to the mitochondria leads to permanent mitochondrial dysfunction and attrition [43,56,57]. Now ATP levels stay low all the time, but the body accommodates to the low ATP level with a decrease in fat mass and a fall in resting energy metabolism. Energy intake must stay low or weight regain will occur.

Consistent with the hypothesis that decreased intracellular ATP is an important trigger involved in obesity and the metabolic syndrome, low ATP can be induced in a variety of cell types and tissues by fructose [16,58–61], as well as by uric acid [12,43,44,62]. Fructose-induced ATP depletion in the liver can be partially reversed by allopurinol [45]. More importantly, a low intracellular ATP state is characteristic of obesity, diabetes, non-alcoholic fatty liver disease (NAFLD) and Alzheimer's disease [25,63–66].

## 4. A description of the ‘survival switch’ induced by fructose metabolism

**Figure 3.**
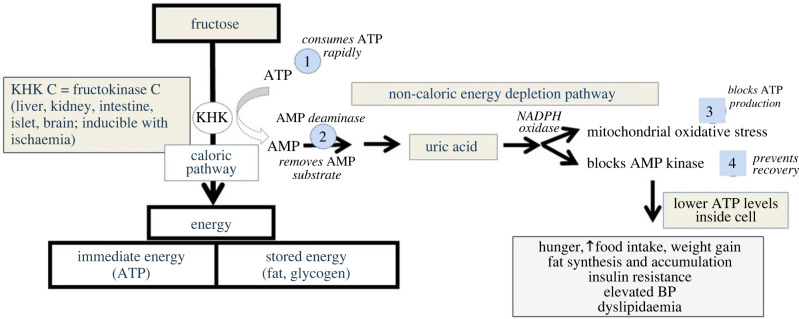
Fructose metabolism and the mechanism for reducing intracellular ATP levels. Fructose is first phosphorylated to fructose-1-phosphate by KHK-C, leading to a rapid consumption of ATP that is directly dependent on the concentration of fructose. Unlike glucose, which never sees a substantial decrease in ATP, the KHK-C driven reaction can plummet both ATP and intracellular phosphate concentrations. In turn, the low intracellular phosphate triggers activation of AMP deaminase-2 (AMPD2). AMP generated during the depletion of ATP is then metabolized to inosine monophosphate (IMP) and eventually to uric acid. The utilization of AMP removes some of the AMP substrate needed to regenerate ATP. However, the intracellular uric acid stimulates NADPH oxidase that translocates to the mitochondria, while simultaneously inhibiting the mitochondrial antioxidant protective mechanisms, particularly Nrf-2. The mitochondrial oxidative stress inhibits both mitochondrial beta fatty acid oxidation (inhibiting enoyl CoA hydratase) and the tricarboxylic acid cycle (by blocking aconitase) resulting in suppression of ATP production by oxidative phosphorylation. In addition, the uric acid also inhibits AMP-activated protein kinase (AMPK) that helps regenerate ATP in low energy states. Thus, the net effect of fructose metabolism KHK-C is that it will reduce intracellular ATP levels. (Online version in colour.)

The administration of fructose can fully replicate the metabolic syndrome, and lead to weight gain, visceral adiposity, insulin resistance, hypertriglyceridaemia, low HDL cholesterol, elevated blood pressure, fatty liver, microalbuminuria, hyperuricaemia and biomarkers of systemic inflammation (including low adiponectin, elevated leptin and elevated high-sensitivity C-reactive protein) [67]. These findings are also observed in animals preparing for hibernation, suggesting that the term ‘metabolic syndrome’ may be a misnomer and that these characteristics instead should be described as a ‘fat-storage syndrome’ [68]. All these features developed as part of a survival response (figure 3) and are described as follows:

### (a) Search for food and water

Fructose encourages behavioural changes to aid the search for food and water. This includes stimulating hunger and bingeing behaviour through the activation of an orexin-hypothalamic circuit, although this does not by itself stimulate increased food intake [69,70]. Fructose also stimulates thirst, possibly by increasing serum osmolality due to a shifting of water into the cell associated with glycogen production [71]. Most importantly, fructose disrupts normal weight regulation by impairing satiety, resulting in excessive food (energy) intake. This requires several weeks to develop and is mediated by central (hypothalamic) leptin resistance [72,73]. Fructose also acts on the brain to stimulate a foraging response that includes the stimulation of exploratory behaviour, impulsivity, and increased locomotor activity [74,75]. Similar effects can be observed by raising uric acid [74,75]. Studies suggest that the effects are mediated by inhibition of insulin-sensitive areas of the brain involved in self-control, recent memory, and deliberation such as the cerebral cortex, the entorhinal cortex, the hippocampus and the posterior cingulate cortex [76,77].

### (b) Increase fat and glycogen stores

In addition to stimulating excessive caloric intake by inducing leptin resistance [72,73], fructose increases intestinal villi length that may facilitate more effective absorption of food [78]. Fructose metabolism also stimulates lipogenesis [38,41,79], impairs beta fatty acid oxidation [57,80], and may reduce lipolysis by adipocytes due to the development of hyperinsulinaemia [81]. Glycogen stores also increase [82–84].

### (c) Energy conservation

While the foraging response requires energy expenditure, this is compensated by a fall in resting energy metabolism likely related to reduced metabolism secondary to both the blockade of fatty acid oxidation and the effects of insulin resistance to reduce glucose metabolism in muscle [85]. In addition, the reduced glucose uptake by the skeletal muscle and adipocyte has the consequence of preserving blood glucose levels that may remain normal or high, thereby protecting those regions of the brain that are less dependent on insulin for glucose uptake [86]. Nevertheless, some regions of the brain are more dependent on insulin, especially those areas that are involved in inhibiting the foraging response [74]. Fructose impairs insulin signalling to areas [87] that are involved in self-control, deliberation and recent memory [76,77]. The consequence is a stimulation of foraging while energy conservation is assisted by both the systemic and cerebral insulin resistance [75,88].

### (d) Preservation of key body functions

**Figure 4.**
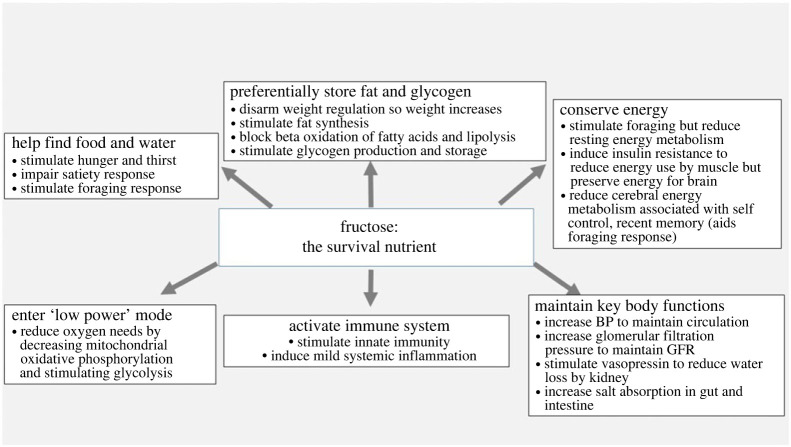
The survival response to fructose.

Animals in precarious conditions need to maintain robust circulation and excretory function. Not surprisingly, fructose metabolism results in an acute increase in blood pressure that is dependent on uric acid [89,90]. Kidney function is maintained by increasing glomerular filtration pressure, and similar findings have been observed with experimental hyperuricaemia [91,92]. Fructose also stimulates vasopressin production [93,94], which can help reabsorb water, and fructose also stimulates sodium absorption both in the kidney proximal tubule [95,96] and in the gut [97]. These effects aid survival (figure 4).

### (e) Activation of the immune system

The generation of uric acid by fructose is associated with the stimulation of inflammatory pathways, including the activation of MAP kinases (p38), NF-ΚB and the stimulation of inflammasomes [98], as well as assisting dendritic cell function [99,100]. Elevated uric acid stimulates oxidative stress [37,101] as well as the production of chemokines, C-reactive protein, and inflammatory pathways [102,103]. Again, immune activation provides protection from infections.

### (f) Entering low power mode

The reduction in mitochondrial oxidative phosphorylation with stimulation of glycolysis induced by fructose metabolism places the body in low power mode and reduces its oxygen needs [17]. This provides an initial benefit in areas of inflammation with local ischaemia, whereas long-term it might drive inflammation and fibrosis. For example, with cardiac ischaemia, there is the production of fructose with inhibition of mitochondrial oxidative phosphorylation and stimulation of glycolysis that drives cardiac remodelling [104]. This appears to be dependent in part on the activation of the transcription factor, HIF-1*α* [104,105]. Reduced mitochondrial function with stimulation of glycolysis is also characteristic of diabetic kidney disease [106,107]. We have reported that diabetic kidney disease is mediated by the conversion of glucose to fructose in the kidney [108]. It seems possible that the benefit of SGLT2 inhibitors in cardiac and renal disease may be by blocking the glucose uptake into the diseased tissues, thereby preventing its subsequent conversion to fructose [109]. The blockade in fructose metabolism may initiate fat oxidation and AMP-activated protein kinase activity similar to that observed in estivating animals [80,110]. The effect of fructose to decrease mitochondrial oxidative phosphorylation and stimulate glycolysis (Warburg effect) provides an explanation for why cancer cells prefer fructose as a growth media [111], and why cancer cell growth is stimulated by both fructose and uric acid [112,113].

## 5. Role of fructose metabolism in the obesity and metabolic syndrome epidemics

The epidemics of obesity and diabetes began in the early twentieth century [114,115]. One likely mechanism is related to the dramatic rise in intake of sugar, with a sharp inflection when HFCS was introduced [115,116]. Soft drinks, and other liquids containing fructose, are particularly effective at activating the pathway as the fall in ATP is directly related to the concentration the hepatocyte is exposed to, which will relate not only to the amount of fructose but also the speed of ingestion [26]. Indeed, we found that slowing down the intake of fructose-containing apple juice could reduce activation of the survival pathway [27]. Indeed, sugary beverage intake correlates with the risk for obesity and metabolic syndrome. Likewise, the global obesity and diabetes epidemic correlates with the rise in sugar intake and with the introduction of sugar into non-western cultures [117]. In addition, the increased intake of processed foods rich in sugar and salt as well as intake of high glycaemic carbs and alcohol also contribute to the amount of fructose ingested or endogenously produced and the risk for obesity, type 2 diabetes and hypertension [118].

However, there is evidence that humans are more sensitive to fructose, as mice and rats are often relatively resistant to the effects of fructose unless large doses are given. In this regard, our group has identified two genetic mutations that markedly enhanced the risk for fructose-induced metabolic syndrome. The first was the mutation in vitamin C that occurred approximately 61 Ma [119] and that was relatively close in time to the asteroid impact that ended the Cretaceous Period [120]. Vitamin C is an antioxidant that can block fructose effects and has been found to have beneficial effects on various components of the metabolic syndrome [121]. Our group has found that fructose-fed vitamin C deficient mice show a dose response in which higher doses of vitamin C result in less obesity for the same amount of fructose ingested (unpublished observation). Thus, the vitamin C mutation may have provided a natural selection advantage to early primates trying to survive during the ‘impact winter’ that occurred following the asteroid collision [122].

The other mutation that likely benefited our primate ancestors was the uricase mutation, that occurred stepwise from around 24 Ma until the gene was fully extinguished in the mid Miocene. Uricase is an enzyme that degrades uric acid, and uricase-deficient mice show a dramatically greater rise in uric acid in response to fructose [123]. We have discussed the evidence that the loss of uricase likely represented a true ‘thrifty gene’ [124]. The mutation occurred during a period of near extinction of ancestral apes from global cooling [125]. Evidence that it might be a thrifty gene was shown by the fact that inhibiting uricase can markedly amplify the ability of fructose to induce metabolic syndrome in rats [126], while resurrecting the ancestral uricase was shown to block the lipogenic effects of fructose in human hepatocytes [127]. Of note, when the mutation occurred, it only doubled serum uric acid levels (to ≈3–4 mg dl^−1^) and thereby acted primarily to protect against starvation as opposed to driving obesity [128]. However, during the twentieth century, serum uric acid levels rose dramatically in parallel with increasing sugar intake and the rise in obesity, diabetes and cardiovascular disease [128]. People with uric acid levels greater than 8 mg dl^−1^ are much more likely to develop obesity and metabolic syndrome, and experimental and pilot studies suggest that the uric acid is playing a contributory role [124].

## 6. How does fructose cause weight gain?

The observation that fructose stimulates food intake as well as lowers resting energy metabolism suggests that both increased energy intake and reduced energy expenditure could be responsible for causing obesity, as determined by gain in body weight and fat mass. However, the reduction in resting energy expenditure is compensated in part by the increased energy used in the foraging response.

To evaluate the importance of both mechanisms, we conducted paired feeding studies in which rats were given foods high in fructose (and in some cases, sugar) and compared with diets of the same composition and caloric content except that the fructose was replaced by starch [129–132]. In some studies, animals were placed on a caloric restriction but with equivalent calories ingested per group [132]. The primary finding was that animals fed the same number of calories showed similar changes in weight whether the diet contained fructose or not. While there was a tendency for a slight increase in weight in the animals fed sugar or fructose (which was likely due to the reduced resting energy metabolism), the overall changes in weight were not significant [132]. Thus, weight gain is primarily accounted for by increased caloric intake [129–132], at least in studies of four months or less in duration.

To identify what drives the increased energy intake, we performed additional studies. First, we found that sweet taste encouraged fructose intake, but mice that lacked taste still preferred fructose compared to water and still became fat (even more so than controls) over time [19]. The preference for fructose was dependent on the metabolism of fructose, as mice lacking fructokinase showed minimal liking of fructose, although they still liked sucrose, likely due to its glucose content [19,133]. Importantly, the preference for fructose was not what drove increased caloric content, based on our studies in which we knocked out fructokinase in specific organs. Indeed, the preference for fructose was mediated by intestinal fructokinase, while the metabolic syndrome was driven by hepatic fructose metabolism. Importantly, liver-specific KHK KO mice would drink large amounts of fructose but were completely protected from both weight gain and the metabolic syndrome [14].

The primary mechanism for increased energy intake was the development of leptin resistance. Initially, animals fed sucrose or fructose reduced their chow intake to maintain neutral energy balance so weight gain would not occur. However, after several weeks the animals increased their chow intake such that total caloric intake became hypercaloric, and this correlated with weight gain. Studies documented that this was due to the development of central leptin resistance, and that it could be prevented by blocking fructose metabolism [72,73]. Indeed, we found that this mechanism was specifically mediated by the fructokinase C isoform, and that it was mediated by the side-chain reaction driving energy depletion [129,134] and unpublished).

Animals preparing for hibernation show similar features of the survival switch (e.g. hyperphagia, leptin resistance, weight gain and fat accumulation, fatty liver and insulin resistance) but shortly before they hibernate they reduce their food intake even though food may still be available and this is associated with a decrease in their metabolism [135,136]. One likely mechanism may relate to reaching a weight that triggers biologic response to reduce food intake (the gravitostat) [137,138]. While this mechanism still occurs in obese humans [139], it is apparent that it can be overrun, which we speculate may relate to continued exposure to high concentrations of fructose.

### (a) Role of fat

Leptin resistance appears to be driven by either ingested or endogenous fructose, whereas other foods such as high fat diets do not cause leptin resistance [72,73]. While fructose mediates leptin resistance, weight gain is amplified by the intake of energy dense, high fat foods in animals who have become leptin resistant [72,73].

Indeed, the blocking of leptin signalling stimulates the preference for high fat foods [140], which makes sense as it would favour more rapid weight gain and fat accumulation. Ground squirrels preferentially eat seeds that contain high polyunsaturated fats when they are actively gaining weight before hibernation [141]. In addition, recently, it was shown that there is a taste for fat mediated by the TRPM5 taste receptor [142] as well as gut-vagal pathways that drive fat preference [143]. This is why the combination of fat with fructose or sugar dramatically increases fat accumulation and weight gain more than either fat or fructose alone [144]. Furthermore, people on a western diet are likely eating sufficient amounts of sugar, salt, high glycaemic carbohydrates and alcohol to induce some leptin resistance in most individuals. We reported, for example, that endogenous fructose could drive ageing changes in mice fed a carbohydrate diet similar to a western diet (50% carbohydrates) despite low sugar content—mice lacking fructokinase remained lean and healthy [145]. These findings also explain why the Inuit remained lean despite a high fat, high protein diet [146], and why ‘low carb’ diets do not cause weight gain despite high fat content. Lard, for example, does not cause weight gain in normal mice unless they are first made leptin resistant with fructose [73].

### (b) Role of glucose

Most of the fructose we ingest is from added sugars (sucrose and HFCS) that also contain glucose. Glucose can markedly enhance fructose absorption and thereby accentuate fructose effects [147,148]. Nevertheless, there is also evidence that glucose administered alone can also induce obesity and metabolic syndrome [7]. A popular hypothesis is that added sugars and high glycaemic carbohydrates may cause obesity by excessive stimulation of insulin [149], which not only stimulates the storage of fat but also blocks lipolysis in the adipocyte [81]. However, as mentioned earlier, high glycaemic foods can also activate the polyol pathway and generate endogenous fructose [7,14].

To evaluate the role of the glucose–insulin axis in driving obesity, we administered glucose to KHK-KO mice or control mice [7]. The control mice showed remarkable weight gain with fatty liver and insulin resistance, but also had high levels of fructose in their livers. Mice lacking KHK drank the same amount of glucose but ate less chow and while they developed mild obesity, it was approximately half that observed in the wild-type controls. The KHK-KO mice also had minimal fatty liver or insulin resistance. These studies thus documented that glucose likely causes some obesity from repeated stimulation of insulin, but that a major mechanism by which high glycaemic carbohydrates cause obesity and metabolic syndrome is via endogenous generation of fructose. Further support came later when we administered HFCS to mice lacking KHK and found that it provided even more protection from obesity, fatty liver and insulin resistance, with about one-quarter of the effect from the glucose–insulin pathway [14]. Thus, while the carbohydrate–insulin model remains an important mechanism for driving obesity the conversion of fructose has an important role in how high glycaemic carbohydrates cause obesity. Interestingly, the insulin resistance induced by fructose results in high fasting insulin levels that act to inhibit lipolysis as the adipocyte remains sensitive to the anti-lipolytic effects of insulin in the insulin resistant state [81]. Thus, the insulin resistant state induced by fructose also supports the glucose–insulin hypothesis but has endogenous fructose as an intermediary step.

### (c) Role of protein

Protein is critical to maintain lean body mass, and there is some evidence that low protein diets may stimulate increased energy intake to accomplish this goal, but with the consequence of increasing body fat stores (protein leverage hypothesis) [150]. However, most low protein diets are high carbohydrate diets making it difficult to know which is more important in driving obesity, and low protein diets also stimulate FGF21 which counters the effects of fructose by stimulating the oxidation of fat, especially in the liver [151].

Furthermore, certain proteins, such as red meats and processed meats, may confer increased risk for diabetes, obesity, gout and chronic kidney disease [152–157]. One likely explanation is that red meats and shellfish are high in glutamate and nucleotides such as IMP and AMP [158]. IMP is a key component of the fructose energy depletion pathway, and glutamate is also metabolized to uric acid in the liver. Indeed, we found that umami (both glutamate and IMP) could generate uric acid and cause hepatic ATP depletion, leptin resistance and obesity and metabolic syndrome similar to fructose [12]. Blocking uric acid production with allopurinol could block the effect of monosodium glutamate to cause obesity [12]. The mechanism by which glutamate stimulates uric acid may relate to the conversion to glutamine and de novo synthesis of uric acid [159,160]. It is known that gouty subjects tend to have high serum glutamate levels and also develop gout from glutamate-rich foods (such as tomatoes) [161,162]. However, most purine-rich vegetables appear to be less likely to raise uric acid and cause gout [163], likely because the purines tend to be relatively poor in AMP and IMP [158].

It is interesting that the purine- and glutamate-rich foods that raise uric acid tend to be umami foods that have their own taste, suggesting that these are foods animals tend to seek [164,165]. While uric acid has a direct role in activating the switch through its ability to stimulate mitochondrial oxidative stress and inhibit AMPK [41,47], it also can stimulate fructose production and enhance KHK expression [13,166]. Thus, much of the effect is by stimulating fructose production from glucose, which likely explains why high protein diets in the absence of carbohydrates do not cause obesity.

## 7. Fructose and metabolic syndrome: an effect independent of excessive calories

A remarkable effect of our pair-feeding studies was the observation that, while weight gain was driven by excessive calories, other effects of fructose occurred even when caloric intake was restricted [129–131]. Indeed, in one study in which rats were fed a hypocaloric, high sucrose diet, the sucrose-fed rats still developed severe fatty liver, hypertriglyceridaemia, insulin resistance and elevations in blood pressure [132]. We also found that the rats initially developed hyperinsulinaemia with normal blood glucose levels, but over time they showed progressive falls in insulin associated with the development of overt diabetes. The pancreatic islets also showed hyalinosis and inflammation, with de novo expression of urate transporters on the islet cells. The starch-fed control animals also developed some signs of mild hypertriglyceridaemia and insulin resistance, which we now believe is likely from low grade fructose generation. However, the overall differences between the two groups were striking [132]. These studies confirm that metabolic syndrome, including alterations in body composition that lead to increased fat, can be associated with minimal weight gain. Indeed, NAFLD can occur in lean individuals [167], especially if hyperuricaemia is present [41].

## 8. The unexpected role of salt, dehydration and vasopressin in obesity

Water is a critical resource, and it is not surprising that fructose increases vasopressin blood levels as a potential mechanism to increase the retention of water by vasopressin-dependent concentration of the urine [93,94,168]. We also found that fructose may have a fundamental role in the regulation of vasopressin, for dehydration (hyperosmolarity) was found to induce activation of the polyol pathway in the supraoptic nucleus in association with an increase in vasopressin synthesis, and KHK-KO mice showed a blunted vasopressin response to acute and chronic dehydration [169].

The big surprise was that vasopressin does more than stimulate water reabsorption in the kidney, but that it is downstream of fructose where it drives most of the features of the survival switch. For years it had been known that there is another vasopressin receptor known as the V1b receptor, but its physiological function was not well understood. We found that the V1b receptor knockout mouse was completely protected from fructose-induced metabolic syndrome [93]. While the exact mechanism is not fully understood, we do know that the V1b receptor stimulates adrenocorticotropic hormone (ACTH) and glucagon levels, and that it may also regulate KHK expression [93].

In retrospect, it has been known that fat is used by marine and desert mammals as a source of metabolic water generation [170,171]. Animals depend on metabolic water when they are hibernating, estivating, as do long-distance migrating birds. The ingestion of salt can induce hyperosmolarity and a dehydration-like state without the loss of water, and this can also activate the production of fructose and vasopressin and cause obesity and metabolic syndrome in laboratory mice that is dependent on fructokinase [10]. Salt intake is high in subjects with obesity and predicts the development of metabolic syndrome, diabetes and fatty liver [10]. Most people with obesity also show signs of dehydration and have high vasopressin levels (noted by measuring serum copeptin) [172–174]. Hydration with water to suppress vasopressin levels can also partially stop and even reverse features of metabolic syndrome [10]. These studies suggest that the survival switch is intricately linked with a nutrient (fructose), a metabolic waste product (uric acid) and a hormone (vasopressin), and that obesity truly is a hormonal disorder.

## 9. The short and long-term health consequences of the survival switch

One of the most serious consequences of continued activation of the fructose-mediated switch is not obesity and weight gain, but the metabolic effects that involve many of the common diseases occurring in western society (table 1) [16,18,25,38,41, 47,74,75,91,92,111,132,145,175–184]. This not only includes classic diseases associated with obesity such as diabetes, NAFLD and gout, but also hypertension, coronary artery disease, certain cancers, behavioural disorders and dementia.

**Table 1.** Overactivation of the survival switch may drive metabolic diseases.

survival feature	disease	initial reversible mechanism	chronic less-reversible mechanism	references
weight gain and fat accumulation	visceral obesity	leptin resistance, ↑intestinal absorptive area, mitochondrial oxidative stress, lipogenesis, impaired fat oxidation	adipocyte senescence and mitochondria loss may lead to persistent obese state	[38,41,47,72,78,175]
systemic insulin resistance and islet dysfunction	type 2 diabetes	systemic insulin resistance and impaired insulin secretion	loss of islets from oxidative injury and scarring leads to persistent diabetic state	[132]
elevation of blood pressure	primary hypertension	uric acid-induced hypertension due to endothelial dysfunction, oxidative stress and angiotensin II	kidney-dependent hypertension associated with inflammation and salt-sensitivity	[176,177]
fat deposition in liver	nonalcoholic fatty liver disease	fat deposition with mild inflammation	cirrhosis	[25,178]
increase in lipids in blood	dyslipidaemia	increased risk for fat deposition in blood vessels	atherosclerosis	[179]
preservation of kidney excretion	chronic kidney disease	uric acid-dependent hyperfiltration and elevated glomerular filtration pressure	progressive nephron loss from arteriolopathy, inflammation and hyperfiltration	[91,92,180]
elevations in uric acid (part of general survival response)	gout, CKD and heart disease	gout and urate crystal deposition in blood vessels and kidney, systemic inflammation	crystal deposition leads to local injury and scarring, atherosclerotic plaques, vascular calcification	[181,182]
protect from hypoxia (mitochondrial suppression with stimulation of glycolysis)	obesity-associated cancers	stimulate cancer growth by allowing cells to better survive in hypoxic environments before blood supply is established	cancer metastases (especially breast, colon, pancreatic)	[111]
	activated at sites of tissue ischaemia (kidney, heart)	local oxidative stress and inflammation exacerbate local tissue injury	cardiac remodelling, kidney tissue scarring	[16,183]
foraging behaviour	behavioural disorders (ADHD, bipolar disease, gambling), Alzheimer's disease	decreased cerebral glucose metabolism in insulin-dependent areas associated with self-control, deliberation and recent memory	mitochondrial loss, neuronal loss	[74,75,88]
recurrent activation of the switch	accelerated ageing	low grade inflammation, mitochondrial suppression, decreased Nrf2, sirtuins	mitochondria loss, capillary loss, low grade scarring	[145]
pregnancy	preeclampsia	ischaemia drives continued fructose generation in placenta and fetus	increased long-term risk for hypertension and kidney disease	[18]
chronic stimulation of ACTH via V1b receptor	aldosteronism	aldosterone breakthrough, aldosterone effects in resistant hypertension	development of primary aldosteronoma	[184]

An example is the emerging role for the pathway in driving coronary artery disease. Recently, it has been recognized that systemic inflammation as well as inflammation in the atherosclerotic plaques play a role in coronary artery disease and myocardial infarction [185]. One potential mechanism is from the effects of soluble uric acid to activate inflammasomes and NFkB-mediated inflammation [98,102]. However, a more dramatic mechanism has recently been identified, as 85% of subjects with gout have uric acid crystals in their blood vessels where it appears to be concentrated in the atherosclerotic plaque [181,186]. Indeed, hyperuricaemia and gout are associated with increased cardiovascular mortality by both epidemiological studies and Mendelian randomization studies [187,188].

Another example is ageing. Fructose metabolism is associated with oxidative stress, mitochondrial dysfunction, loss of cytoprotective transcription factor nuclear factor erythroid 2-related factor 2 (Nrf2) and a reduction in sirtuins that characterize the ageing process [50,189]. Fructose also induces the generation of advanced glycation endproducts much more effectively than glucose [190,191]. As mentioned, we found that ageing-associated kidney disease did not occur in ageing KHK-KO mice [145].

There is also increasing evidence that Alzheimer's disease may be related to intracerebral fructose metabolism [75]. Alzheimer's disease is increased in those whose diet is enriched in foods that either contain or produce fructose (high sugar, high salt, high glycaemic and high processed red meat diets) or who have features of metabolic syndrome and diabetes. The administration of fructose can also induce features of Alzheimer's in laboratory rats (including decreased mental status and amyloid and τ protein deposition), and fructose is also elevated in the brains of individuals with early Alzheimer's dementia (reviewed in [75,88]). The mechanism appears to be secondary to the biological effects of excessive fructose to suppress certain regions of the brain to stimulate a foraging response [75,88].

One key finding is that the role of the fructose pathway is almost inevitably strongest in early disease states, for over time there is often fibrosis, inflammation or mitochondrial loss that results in persistence of the disease process (table 1). Thus, the best time for intervention may turn out to be in early disease before conditions become less reversible. Nevertheless, since much of the disease represents a metabolic disorder in which mitochondrial function is inhibited, one ideal approach is to try to regenerate mitochondria by exercise or other means. In this regard, while both low fructose and low salt diets improve mitochondrial function [192], another excellent approach is via exercise [23].

## 10. Limitations and challenges

There are challenges to the hypothesis. First, there has been a dispute over whether the thrifty gene hypothesis exists, as it seems most famines are too short to provide sufficient natural selection pressures to result in the emergence of a survival gene that would determine the fate of a species (discussed in [124,193]). Some have also argued that if thrifty genes existed, then everyone today should be obese. However, the mutations in uricase occurred during seasonal famine that lasted millions of years, and the mutation did not cause obesity but rather prevented starvation. Our hypothesis also suggests that obesity will not occur in everyone as it requires an interaction in which one becomes leptin resistant (from fructose metabolism) and then ingests high energy foods (such as fat).

We agree that during the period of modern humans, it is much less likely for a survival mutation to arise. Having said this, there was a period of 5 000–10 000 years during the Ice Age in which advancing glaciers and reduced big game availability led to episodic starvation and a contraction in the population that influenced the development of shamanism and art [194]. There was also a 200-year drought around 4.2 K BP that led to the fall of multiple kingdoms and resulted in the rapid spread of the lactase persistence gene where it carried survival advantages [195]. However, survival genes are much less likely to occur now, and increased fat stores are much less likely to confer benefit, and so it is more likely that today obesity genes may spread more by random drift (the drifty gene hypothesis [196]), especially since predation is less of a risk for those who are obese and sedentary in a civilized society [196,197].

There is also some evidence that intake of sugary beverages have decreased in the last decade, resulting in less overall intake of added sugars [198]. However, intake of added sugars remains extensive and still accounts for 15 per cent of overall dietary intake. Processed foods still contain excessive added sugars and salt in over 70 per cent of the products [199,200]. Thus, it is likely that exposure to foods that either contain or stimulate fructose production remains over the threshold necessary to induce leptin resistance in most individuals.

There is also some evidence that fats may drive obesity on their own in the absence of fructose. We have noted that saturated fats (butter) can induce some modest obesity in our mice lacking fructokinase, and this was associated with mild liver steatosis [144]. Saturated fats appear to be able to induce hepatic steatosis much better than other fats due to their ability to inhibit lipolysis and increase free fatty acid delivery to the liver [201]. Another study found that diets high in animal fat (especially butter and cheese) might increase the risk for diabetes [202]. Nevertheless, the lack of obesity and diabetes in the Inuit on their diet of meats and saturated fat make us think that this is not a dominant mechanism driving obesity and insulin resistance. Indeed, intake of saturated fats fell last century and does not correlate with the epidemics of obesity, diabetes and NAFLD [203], whereas NAFLD correlates with sugary beverage intake [204].

Another confusing finding is that subjects on a low carb, ketogenic diet often develop hyperuricaemia resulting from ketonuria that interferes with uric acid excretion. While the high uric acid can occasionally precipitate gout, it is not known whether the hyperuricaemia has any other deleterious effects. There is some thought that it might be present to help stimulate gluconeogenesis from amino acids [48], but it is also possible that it is not inflammatory due to the countering effects of ketonaemia [205] or because uric acid-induced fructose generation is less likely in the setting of a low carbohydrate diet. We do know that in the situation of starvation, and quite possible severe carbohydrate restriction, that fructose no longer stimulates fat storage but is rather preferentially converted to glucose for immediate energy needs [52]. Clearly, more studies need to be done to address the role of hyperuricaemia in people on a ketogenic diet.

Finally, it is known that not all hibernating animals ingest fructose as their triggering event to increase their food intake, and some animals in hibernacula may eat the same chow yet still undergo an apparent switch in which they dramatically increase their fat stores. It is not clear what is the trigger in these animals, although it could relate to alterations in hydration status, food insecurity, genetic factors or other unknown mechanisms [206–208]. We do know that ground squirrels that enter hibernation despite being on the same diet nevertheless show metabolic changes in their liver similar to that observed with fructose metabolism, in which weight gain is associated with activation of AMP deaminase and uric acid in the liver, followed by stimulation of AMP-activated protein kinase when they oxidize fat during hibernation [80]. Clearly, more studies are needed.

## 11. Testable predictions

While there remains a lot of supportive evidence for the fructose survival hypothesis, there are also testable predictions. First, there is a major need to confirm the importance of the endogenous fructose pathway in the human population, and to determine the magnitude of the effect on biological outcomes. One potential way is to develop drugs that can interfere with the pathway, such as to develop effective inhibitors of KHK, AMP deaminase-2, or other enzymes in the survival pathway. Studies to investigate the role of the endogenous fructose pathway in hibernating mammals and long-distance migrating birds would also be helpful. A second step is to better understand the mechanisms driving the transition from an early more reversible state to one that is more permanent. One way is to identify biomarkers that document this transition. For example, the importance of the vasopressin pathway can be tested most effectively by assessing serum copeptin as part of the initial workup of a subject at risk for metabolic syndrome and to perform a study in which it is either inhibited by hydration and salt and sugar restriction or inhibited using a vasopressin 1b receptor blocker [93]. Likewise, studies investigating the role of uric acid might be most likely to show benefit on metabolic features early in the course of the disease [176,209].

## 12. Summary

Obesity and its associated metabolic diseases have had devastating health consequences on modern society. Here, we link these diseases with the activation of a major survival pathway developed in nature to help animals prepare for times of scarcity. Unfortunately, we have unknowingly adopted the foods that activate this switch in our everyday diet, and coupled with the thrifty genes we picked up, we are now suffering the consequences of putting this survival pathway in overdrive. The tragedy of our success is even greater than thought, for newer studies suggest that the fructose pathway may also increase our risk for cancer, pregnancy-related disease and neurological disorders. We recommend proceeding with studies such as outlined above to better understand the role of fructose metabolism in health and disease.

## Data Availability

This article has no additional data.

## References

[1] Benner S. 2017 Uniting natural history with the molecular sciences. The ultimate multidisciplinarity. Acc. Chem. Res. **50**, 498-502. (10.1021/acs.accounts.6b00496) 28945399

[2] Benner SA, Caraco MD, Thomson JM, Gaucher EA. 2002 Planetary biology—paleontological, geological, and molecular histories of life. Science **296**, 864-868. (10.1126/science.1069863) 11988562

[3] Stenvinkel P, Painer J, Johnson RJ, Natterson-Horowitz B. 2020 Biomimetics—nature's roadmap to insights and solutions for burden of lifestyle diseases. J. Intern. Med. **287**, 238-251. (10.1111/joim.12982) 31639885 PMC7035180

[4] Abegglen LM et al. 2015 Potential mechanisms for cancer resistance in elephants and comparative cellular response to DNA damage in humans. J. Am. Med. Assoc. **314**, 1850-1860. (10.1001/jama.2015.13134) PMC4858328 26447779

[5] Neel JV. 1962 Diabetes mellitus: a ‘thrifty’ genotype rendered detrimental by ‘progress’? Am. J. Hum. Genet. **14**, 353-362. 13937884 PMC1932342

[6] Yang Q, Zhang Z, Gregg EW, Flanders WD, Merritt R, Hu FB. 2014 Added sugar intake and cardiovascular diseases mortality among US adults. JAMA Intern. Med. **174**, 516-524. (10.1001/jamainternmed.2013.13563) 24493081 PMC10910551

[7] Lanaspa MA et al. 2013 Endogenous fructose production and metabolism in the liver contributes to the development of metabolic syndrome. Nat. Commun. **4**, 2434. (10.1038/ncomms3434) 24022321 PMC3833672

[8] Francey C, Cros J, Rosset R, Creze C, Rey V, Stefanoni N, Schneiter P, Tappy L, Seyssel K. 2019 The extra-splanchnic fructose escape after ingestion of a fructose-glucose drink: an exploratory study in healthy humans using a dual fructose isotope method. Clin. Nutr. ESPEN **29**, 125-132. (10.1016/j.clnesp.2018.11.008) 30661675

[9] Hwang JJ, Jiang L, Hamza M, Dai F, Belfort-Deaguiar R, Cline G, Rothman DL, Mason G, Sherwin RS. 2017 The human brain produces fructose from glucose. JCI Insight **2**, e90508. (10.1172/jci.insight.90508) 28239653 PMC5313070

[10] Lanaspa MA et al. 2018 High salt intake causes leptin resistance and obesity in mice by stimulating endogenous fructose production and metabolism. Proc. Natl Acad. Sci. USA **115**, 3138-3143. (10.1073/pnas.1713837115) 29507217 PMC5866545

[11] Wang M, Chen WY, Zhang J, Gobejishvili L, Barve SS, Mcclain CJ, Joshi-Barve S. 2020 Elevated fructose and uric acid through aldose reductase contribute to experimental and human alcoholic liver disease. Hepatology **72**, 1617-1637. (10.1002/hep.31197) 32086945

[12] Andres-Hernando A, Cicerchi C, Kuwabara M, Orlicky DJ, Sanchez-Lozada LG, Nakagawa T, Johnson RJ, Lanaspa MA. 2021 Umami-induced obesity and metabolic syndrome is mediated by nucleotide degradation and uric acid generation. Nat. Metab **3**, 1189-1201. (10.1038/s42255-021-00454-z) 34552272 PMC9987717

[13] Sanchez-Lozada LG, Andres-Hernando A, Garcia-Arroyo FE, Cicerchi C, Li N, Kuwabara M, Roncal-Jimenez CA, Johnson RJ, Lanaspa MA. 2019 Uric acid activates aldose reductase and the polyol pathway for endogenous fructose and fat production causing development of fatty liver in rats. J. Biol. Chem. **294**, 4272-4281. (10.1074/jbc.RA118.006158) 30651350 PMC6422088

[14] Andres-Hernando A, Orlicky DJ, Kuwabara M, Ishimoto T, Nakagawa T, Johnson RJ, Lanaspa MA. 2020 Deletion of fructokinase in the liver or in the intestine reveals differential effects on sugar-induced metabolic dysfunction. Cell Metab. **32**, 117-127. (10.1016/j.cmet.2020.05.012) 32502381 PMC7347444

[15] Mirtschink P et al. 2015 HIF-driven SF3B1 induces KHK-C to enforce fructolysis and heart disease. Nature **522**, 444-449. (10.1038/nature14508) 26083752 PMC4783869

[16] Andres-Hernando A et al. 2017 Protective role of fructokinase blockade in the pathogenesis of acute kidney injury in mice. Nat. Commun. **8**, 14181. (10.1038/ncomms14181) 28194018 PMC5316807

[17] Park TJ et al. 2017 Fructose-driven glycolysis supports anoxia resistance in the naked mole-rat. Science **356**, 307-311. (10.1126/science.aab3896) 28428423

[18] Nakagawa T et al. 2023 Fructose might be a clue to the origin of preeclampsia. Insights from nature and evolution. Hypertens. Res. **46**, 646-653. (10.1038/s41440-022-01121-w) 36539464 PMC10015507

[19] Andres-Hernando A, Kuwabara M, Orlicky DJ, Vandenbeuch A, Cicerchi C, Kinnamon SC, Finger TE, Johnson RJ, Lanaspa MA. 2020 Sugar causes obesity and metabolic syndrome in mice independently of sweet taste. Am. J. Physiol. Endocrinol. Metab. **319**, E276-E290. (10.1152/ajpendo.00529.2019) 32574112 PMC7473911

[20] Keesey RE, Powley TL. 2008 Body energy homeostasis. Appetite **51**, 442-445. (10.1016/j.appet.2008.06.009) 18647629 PMC2605663

[21] Bessesen DH. 2011 Regulation of body weight: what is the regulated parameter? Physiol. Behav. **104**, 599-607. (10.1016/j.physbeh.2011.05.006) 21565211

[22] Blundell JE, Finlayson G, Gibbons C, Caudwell P, Hopkins M. 2015 The biology of appetite control: do resting metabolic rate and fat-free mass drive energy intake? Physiol. Behav. **152**, 473-478. (10.1016/j.physbeh.2015.05.031) 26037633

[23] Waters DL, Brooks WM, Qualls CR, Baumgartner RN. 2003 Skeletal muscle mitochondrial function and lean body mass in healthy exercising elderly. Mech. Ageing Dev. **124**, 301-309. (10.1016/s0047-6374(02)00197-5) 12663127

[24] Bawden SJ, Stephenson MC, Ciampi E, Hunter K, Marciani L, Macdonald IA, Aithal GP, Morris PG, Gowland PA. 2016 Investigating the effects of an oral fructose challenge on hepatic ATP reserves in healthy volunteers: a (31)P MRS study. Clin. Nutr. **35**, 645-649. (10.1016/j.clnu.2015.04.001) 25935852

[25] Abdelmalek MF et al. 2012 Higher dietary fructose is associated with impaired hepatic adenosine triphosphate homeostasis in obese individuals with type 2 diabetes. Hepatology **56**, 952-960. (10.1002/hep.25741) 22467259 PMC3406258

[26] Sundborn G, Thornley S, Merriman TR, Lang B, King C, Lanaspa MA, Johnson RJ. 2019 Are liquid sugars different from solid sugar in their ability to cause metabolic syndrome? Obesity **27**, 879-887. (10.1002/oby.22472) 31054268

[27] Kanbay M et al. 2021 The speed of ingestion of a sugary beverage has an effect on the acute metabolic response to fructose. Nutrients **13**, 1916. (10.3390/nu13061916) 34199607 PMC8228203

[28] Maenpaa PH, Raivio KO, Kekomaki MP. 1968 Liver adenine nucleotides: fructose-induced depletion and its effect on protein synthesis. Science **161**, 1253-1254. (10.1126/science.161.3847.1253) 5673437

[29] Van Den Berghe G, Bronfman M, Vanneste R, Hers HG. 1977 The mechanism of adenosine triphosphate depletion in the liver after a load of fructose. A kinetic study of liver adenylate deaminase. Biochem. J. **162**, 601-609. (10.1042/bj1620601) 869906 PMC1164643

[30] Perheentupa J, Raivio K. 1967 Fructose-induced hyperuricaemia. Lancet **2**, 528-531. (10.1016/S0140-6736(67)90494-1) 4166890

[31] Raivio KO, Becker A, Meyer LJ, Greene ML, Nuki G, Seegmiller JE. 1975 Stimulation of human purine synthesis de novo by fructose infusion. Metabolism **24**, 861-869. (10.1016/0026-0495(75)90133-X) 166270

[32] Johnson TA, Jinnah HA, Kamatani N. 2019 Shortage of cellular ATP as a cause of diseases and strategies to enhance ATP. Front. Pharmacol. **10**, 98. (10.3389/fphar.2019.00098) 30837873 PMC6390775

[33] Stirpe F, Della Corte E, Bonetti E, Abbondanza A, Abbati A, De Stefano F. 1970 Fructose-induced hyperuricaemia. Lancet **2**, 1310-1311. (10.1016/S0140-6736(70)92269-5) 4098798

[34] Al-Ozairi E et al. 2020 Fructose tolerance test in obese people with and without type 2 diabetes. J. Diabetes **12**, 197-204. (10.1111/1753-0407.12984) 31472036 PMC7151745

[35] Cantu-Medellin N, Kelley EE. 2013 Xanthine oxidoreductase-catalyzed reactive species generation: a process in critical need of reevaluation. Redox Biol. **1**, 353-358. (10.1016/j.redox.2013.05.002) 24024171 PMC3757702

[36] Sautin YY, Nakagawa T, Zharikov S, Johnson RJ. 2007 Adverse effects of the classic antioxidant uric acid in adipocytes: NADPH oxidase-mediated oxidative/nitrosative stress. Am. J. Physiol. Cell Physiol. **293**, C584-C596. (10.1152/ajpcell.00600.2006) 17428837

[37] Sanchez-Lozada LG, Soto V, Tapia E, Avila-Casado C, Sautin YY, Nakagawa T, Franco M, Rodriguez-Iturbe B, Johnson RJ. 2008 Role of oxidative stress in the renal abnormalities induced by experimental hyperuricemia. Am. J. Physiol. Renal. Physiol. **295**, F1134-F1141. (10.1152/ajprenal.00104.2008) 18701632 PMC2576157

[38] Choi YJ et al. 2014 Uric acid induces fat accumulation via generation of endoplasmic reticulum stress and SREBP-1c activation in hepatocytes. Lab. Invest. **94**, 1114-1125. (10.1038/labinvest.2014.98) 25111690

[39] Ko J et al. 2019 Uric acid induced the phenotype transition of vascular endothelial cells via induction of oxidative stress and glycocalyx shedding. FASEB J. **33**, 13 334-13 345. (10.1096/fj.201901148R) 31553887

[40] Chao HH, Liu JC, Lin JW, Chen CH, Wu CH, Cheng TH. 2008 Uric acid stimulates endothelin-1 gene expression associated with NADPH oxidase in human aortic smooth muscle cells. Acta Pharmacol. Sin. **29**, 1301-1312. (10.1111/j.1745-7254.2008.00877.x) 18954524

[41] Lanaspa MA et al. 2012 Uric acid induces hepatic steatosis by generation of mitochondrial oxidative stress: potential role in fructose-dependent and -independent fatty liver. J. Biol. Chem. **287**, 40 732-40 744. (10.1074/jbc.M112.399899) PMC3504786 23035112

[42] Garcia-Arroyo FE et al. 2021 Osthol ameliorates kidney damage and metabolic syndrome induced by a high-fat/high-sugar diet. Int. J. Mol. Sci. **22**, 2431. (10.3390/ijms22052431) 33670975 PMC7957708

[43] Sanchez-Lozada LG et al. 2012 Uric acid-induced endothelial dysfunction is associated with mitochondrial alterations and decreased intracellular ATP concentrations. Nephron Exp. Nephrol. **121**, e71-e78. (10.1159/000345509) 23235493 PMC3656428

[44] Cristobal-Garcia M et al. 2015 Renal oxidative stress induced by long-term hyperuricemia alters mitochondrial function and maintains systemic hypertension. Oxid. Med. Cell Longev. **2015**, 535686. (10.1155/2015/535686) 25918583 PMC4396880

[45] Garcia-Arroyo FE et al. 2019 Allopurinol prevents the lipogenic response induced by an acute oral fructose challenge in short-term fructose fed rats. Biomolecules **9**, 601. (10.3390/biom9100601) 31614639 PMC6843394

[46] Helsley RN et al. 2023 Ketohexokinase-C regulates global protein acetylation to decrease carnitine palmitoyltransferase 1a-mediated fatty acid oxidation. J. Hepatol., Epub ahead of print. (10.1016/j.jhep.2023.02.010) PMC10679901 36822479

[47] Lanaspa MA et al. 2012 Counteracting roles of AMP deaminase and AMP kinase in the development of fatty liver. PLoS ONE **7**, e48801. (10.1371/journal.pone.0048801) 23152807 PMC3494720

[48] Cicerchi C et al. 2014 Uric acid-dependent inhibition of AMP kinase induces hepatic glucose production in diabetes and starvation: evolutionary implications of the uricase loss in hominids. FASEB J. **28**, 3339-3350. (10.1096/fj.13-243634) 24755741 PMC4101654

[49] Zhao S et al. 2020 Dietary fructose feeds hepatic lipogenesis via microbiota-derived acetate. Nature **579**, 586-591. (10.1038/s41586-020-2101-7) 32214246 PMC7416516

[50] Rodriguez-Iturbe B, Johnson RJ, Lanaspa MA, Nakagawa T, Garcia-Arroyo FE, Sanchez-Lozada LG. 2022 Sirtuin deficiency and the adverse effects of fructose and uric acid synthesis. Am. J. Physiol. Regul. Integr. Comp. Physiol. **322**, R347-R359. (10.1152/ajpregu.00238.2021) 35271385 PMC8993531

[51] Luengo A et al. 2021 Increased demand for NAD(+) relative to ATP drives aerobic glycolysis. Mol. Cell **81**, 691-707. (10.1016/j.molcel.2020.12.012) 33382985 PMC8315838

[52] Gelfand RA, Sherwin RS. 1986 Nitrogen conservation in starvation revisited: protein sparing with intravenous fructose. Metabolism **35**, 37-44. (10.1016/0026-0495(86)90093-4) 3510363

[53] Herman MA, Birnbaum MJ. 2021 Molecular aspects of fructose metabolism and metabolic disease. Cell Metab. **33**, 2329-2354. (10.1016/j.cmet.2021.09.010) 34619074 PMC8665132

[54] Sun SZ, Empie MW. 2012 Fructose metabolism in humans—what isotopic tracer studies tell us. Nutr. Metab. **9**, 89. (10.1186/1743-7075-9-89) PMC3533803 23031075

[55] San-Millan I, Sparagna GC, Chapman HL, Warkins VL, Chatfield KC, Shuff SR, Martinez JL, Brooks GA. 2022 Chronic lactate exposure decreases mitochondrial function by inhibition of fatty acid uptake and cardiolipin alterations in neonatal rat cardiomyocytes. Front. Nutr. **9**, 809485. (10.3389/fnut.2022.809485) 35308271 PMC8931465

[56] Softic S et al. 2017 Divergent effects of glucose and fructose on hepatic lipogenesis and insulin signaling. J. Clin. Invest. **127**, 4059-4074. (10.1172/JCI94585) 28972537 PMC5663363

[57] Softic S et al. 2019 Dietary sugars alter hepatic fatty acid oxidation via transcriptional and post-translational modifications of mitochondrial proteins. Cell Metab. **30**, 735-753. (10.1016/j.cmet.2019.09.003) 31577934 PMC7816129

[58] Tharabenjasin P, Douard V, Patel C, Krishnamra N, Johnson RJ, Zuo J, Ferraris RP. 2014 Acute interactions between intestinal sugar and calcium transport *in vitro*. Am. J. Physiol. Gastrointest. Liver Physiol. **306**, G1-12. (10.1152/ajpgi.00263.2013) 24177030

[59] Cha SH, Wolfgang M, Tokutake Y, Chohnan S, Lane MD. 2008 Differential effects of central fructose and glucose on hypothalamic malonyl-CoA and food intake. Proc. Natl Acad. Sci. USA **105**, 16 871-16 875. (10.1073/pnas.0809255105) PMC2579345 18971329

[60] Cirillo P, Gersch MS, Mu W, Scherer PM, Kim KM, Gesualdo L, Henderson GN, Johnson RJ, Sautin YY. 2009 Ketohexokinase-dependent metabolism of fructose induces proinflammatory mediators in proximal tubular cells. J. Am. Soc. Nephrol. **20**, 545-553. (10.1681/ASN.2008060576) 19158351 PMC2653686

[61] Glushakova O, Kosugi T, Roncal C, Mu W, Heinig M, Cirillo P, Sanchez-Lozada LG, Johnson RJ, Nakagawa T. 2008 Fructose induces the inflammatory molecule ICAM-1 in endothelial cells. J. Am. Soc. Nephrol. **19**, 1712-1720. (10.1681/ASN.2007121304) 18508964 PMC2518440

[62] Shi C, Guo H, Liu X. 2022 High uric acid induced hippocampal mitochondrial dysfunction and cognitive impairment involving intramitochondrial NF-kappaB inhibitor alpha/nuclear factor-kappaB pathway. Neuroreport **33**, 109-115. (10.1097/WNR.0000000000001762) 35139059

[63] Nair SVPC, Arnold C, Diehl AM. 2003 Hepatic ATP reserve and efficiency of replenishing: comparison between obese and nonobese normal individuals. Am. J. Gastroenterol. **98**, 466-470. 12591070 10.1111/j.1572-0241.2003.07221.x

[64] Szendroedi J, Chmelik M, Schmid AI, Nowotny P, Brehm A, Krssak M, Moser E, Roden M. 2009 Abnormal hepatic energy homeostasis in type 2 diabetes. Hepatology **50**, 1079-1086. (10.1002/hep.23093) 19637187

[65] Hoyer S. 2000 Brain glucose and energy metabolism abnormalities in sporadic Alzheimer disease. Causes and consequences: an update. Exp. Gerontol. **35**, 1363-1372. (10.1016/S0531-5565(00)00156-X) 11113614

[66] Hoyer S. 1992 Oxidative energy metabolism in Alzheimer brain. Studies in early-onset and late-onset cases. Mol. Chem. Neuropathol. **16**, 207-224. (10.1007/BF03159971) 1418218

[67] Stanhope KL et al. 2009 Consuming fructose-sweetened, not glucose-sweetened, beverages increases visceral adiposity and lipids and decreases insulin sensitivity in overweight/obese humans. J. Clin. Invest. **119**, 1322-1334. (10.1172/JCI37385) 19381015 PMC2673878

[68] Johnson RJ, Stenvinkel P, Martin SL, Jani A, Sanchez-Lozada LG, Hill JO, Lanaspa MA. 2013 Redefining metabolic syndrome as a fat storage condition based on studies of comparative physiology. Obesity **21**, 659-664. (10.1002/oby.20026) 23401356 PMC3660463

[69] Rorabaugh JM, Stratford JM, Zahniser NR. 2014 A relationship between reduced nucleus accumbens shell and enhanced lateral hypothalamic orexin neuronal activation in long-term fructose bingeing behavior. PLoS ONE **9**, e95019. (10.1371/journal.pone.0095019) 24736531 PMC3988143

[70] Rorabaugh JM, Stratford JM, Zahniser NR. 2015 Differences in bingeing behavior and cocaine reward following intermittent access to sucrose, glucose or fructose solutions. Neuroscience **301**, 213-220. (10.1016/j.neuroscience.2015.06.015) 26079112 PMC4504752

[71] Johnson RJ et al. 2020 Fructose metabolism as a common evolutionary pathway of survival associated with climate change, food shortage and droughts. J. Intern. Med. **287**, 252-262. (10.1111/joim.12993) 31621967 PMC10917390

[72] Shapiro A, Mu W, Roncal C, Cheng KY, Johnson RJ, Scarpace PJ. 2008 Fructose-induced leptin resistance exacerbates weight gain in response to subsequent high-fat feeding. Am. J. Physiol. Regul. Integr. Comp. Physiol. **295**, R1370-R1375. (10.1152/ajpregu.00195.2008) 18703413 PMC2584858

[73] Shapiro A, Tumer N, Gao Y, Cheng KY, Scarpace PJ. 2011 Prevention and reversal of diet-induced leptin resistance with a sugar-free diet despite high fat content. Br. J. Nutr. **106**, 390-397. (10.1017/S000711451100033X) 21418711

[74] Johnson RJ, Wilson WL, Bland ST, Lanaspa MA. 2021 Fructose and uric acid as drivers of a hyperactive foraging response: a clue to behavioral disorders associated with impulsivity or mania? Evol. Hum. Behav. **42**, 194-203. (10.1016/j.evolhumbehav.2020.09.006) 33994772 PMC8117086

[75] Johnson RJ, Gomez-Pinilla F, Nagel M, Nakagawa T, Rodriguez-Iturbe B, Sanchez-Lozada LG, Tolan DR, Lanaspa MA. 2020 Cerebral fructose metabolism as a potential mechanism driving Alzheimer's Disease. Front. Aging Neurosci. **12**, 560865. (10.3389/fnagi.2020.560865) 33024433 PMC7516162

[76] Purnell JQ, Klopfenstein BA, Stevens AA, Havel PJ, Adams SH, Dunn TN, Krisky C, Rooney WD. 2011 Brain functional magnetic resonance imaging response to glucose and fructose infusions in humans. Diabetes Obes. Metab. **13**, 229-234. (10.1111/j.1463-1326.2010.01340.x) 21205113

[77] Page KA et al. 2013 Effects of fructose vs glucose on regional cerebral blood flow in brain regions involved with appetite and reward pathways. J. Am. Med. Assoc. **309**, 63-70. (10.1001/jama.2012.116975) PMC4076145 23280226

[78] Taylor SR et al. 2021 Dietary fructose improves intestinal cell survival and nutrient absorption. Nature **597**, 263-267. (10.1038/s41586-021-03827-2) 34408323 PMC8686685

[79] Softic S et al. 2018 Divergent effects of glucose and fructose on hepatic lipogenesis and insulin signaling. J. Clin. Invest. **128**, 1199. (10.1172/JCI99009) 29493547 PMC5824865

[80] Lanaspa MA et al. 2015 Opposing activity changes in AMP deaminase and AMP-activated protein kinase in the hibernating ground squirrel. PLoS ONE **10**, e0123509. (10.1371/journal.pone.0123509) 25856396 PMC4391924

[81] Tan SX et al. 2015 Selective insulin resistance in adipocytes. J. Biol. Chem. **290**, 11 337-11 348. (10.1074/jbc.M114.623686) PMC4416839 25720492

[82] Matthaei C, Sasse D, Riede UN. 1976 The fructose induced ‘glycogenosis’. II. Histochemical studies of glycogen metabolism in rat liver after fructose overload and similar diets (author's transl). Beitr. Pathol. **157**, 56-75. (10.1016/S0005-8165(76)80148-5) 178297

[83] Conlee RK, Lawler RM, Ross PE. 1987 Effects of glucose or fructose feeding on glycogen repletion in muscle and liver after exercise or fasting. Ann. Nutr. Metab. **31**, 126-132. (10.1159/000177259) 3592616

[84] Youn JH, Kaslow HR, Bergman RN. 1987 Fructose effect to suppress hepatic glycogen degradation. J. Biol. Chem. **262**, 11 470-11 477. (10.1016/S0021-9258(18)60830-0) 3114246

[85] Cox CL et al. 2012 Consumption of fructose-sweetened beverages for 10 weeks reduces net fat oxidation and energy expenditure in overweight/obese men and women. Eur. J. Clin. Nutr. **66**, 201-208. (10.1038/ejcn.2011.159) 21952692 PMC3252467

[86] Seaquist ER, Damberg GS, Tkac I, Gruetter R. 2001 The effect of insulin on *in vivo* cerebral glucose concentrations and rates of glucose transport/metabolism in humans. Diabetes **50**, 2203-2209. (10.2337/diabetes.50.10.2203) 11574399

[87] Agrawal R, Noble E, Vergnes L, Ying Z, Reue K, Gomez-Pinilla F. 2016 Dietary fructose aggravates the pathobiology of traumatic brain injury by influencing energy homeostasis and plasticity. J. Cereb. Blood Flow Metab. **36**, 941-953. (10.1177/0271678X15606719) 26661172 PMC4853835

[88] Johnson RJ, Tolan DR, Bredesen D, Nagel M, Sanchez-Lozada LG, Fini M, Burtis S, Lanaspa MA, Perlmutter D. 2023 Could Alzheimer's disease be a maladaptation of an evolutionary survival pathway mediated by intracerebral fructose and uric acid metabolism? Am. J. Clin. Nutr. **117**, 455-466. (10.1016/j.ajcnut.2023.01.002) 36774227 PMC10196606

[89] Brown CM, Dulloo AG, Yepuri G, Montani JP. 2008 Fructose ingestion acutely elevates blood pressure in healthy young humans. Am. J. Physiol. Regul. Integr. Comp. Physiol. **294**, R730-R737. (10.1152/ajpregu.00680.2007) 18199590

[90] Perez-Pozo SE, Schold J, Nakagawa T, Sanchez-Lozada LG, Johnson RJ, Lillo JL. 2010 Excessive fructose intake induces the features of metabolic syndrome in healthy adult men: role of uric acid in the hypertensive response. Int. J. Obes. **34**, 454-461. (10.1038/ijo.2009.259) 20029377

[91] Sanchez-Lozada LG et al. 2007 Fructose-induced metabolic syndrome is associated with glomerular hypertension and renal microvascular damage in rats. Am. J. Physiol. Renal. Physiol. **292**, F423-F429. (10.1152/ajprenal.00124.2006) 16940562

[92] Sanchez-Lozada LG, Tapia E, Santamaria J, Avila-Casado C, Soto V, Nepomuceno T, Rodriguez-Iturbe B, Johnson RJ, Herrera-Acosta J. 2005 Mild hyperuricemia induces vasoconstriction and maintains glomerular hypertension in normal and remnant kidney rats. Kidney Int. **67**, 237-247. (10.1111/j.1523-1755.2005.00074.x) 15610247

[93] Andres-Hernando A et al. 2021 Vasopressin mediates fructose-induced metabolic syndrome by activating the V1b receptor. JCI Insight **6**, e140848. (10.1172/jci.insight.140848) 33320834 PMC7821599

[94] Chapman CL, Johnson BD, Sackett JR, Parker MD, Schlader ZJ. 2019 Soft drink consumption during and following exercise in the heat elevates biomarkers of acute kidney injury. Am. J. Physiol. Regul. Integr. Comp. Physiol. **316**, R189-R198. (10.1152/ajpregu.00351.2018) 30601706

[95] Cabral PD, Hong NJ, Hye Khan MA, Ortiz PA, Beierwaltes WH, Imig JD, Garvin JL. 2014 Fructose stimulates Na/H exchange activity and sensitizes the proximal tubule to angiotensin II. Hypertension **63**, e68-e73. (10.1161/HYPERTENSIONAHA.113.02564) 24379189

[96] Hayasaki T et al. 2019 Fructose increases the activity of sodium hydrogen exchanger in renal proximal tubules that is dependent on ketohexokinase. J. Nutr. Biochem. **71**, 54-62. (10.1016/j.jnutbio.2019.05.017) 31276916

[97] Soleimani M. 2011 Dietary fructose, salt absorption and hypertension in metabolic syndrome: towards a new paradigm. Acta Physiol. **201**, 55-62. (10.1111/j.1748-1716.2010.02167.x) PMC10624004 21143427

[98] Braga TT et al. 2017 Soluble uric acid activates the NLRP3 inflammasome. Sci. Rep. **7**, 39884. (10.1038/srep39884) 28084303 PMC5233987

[99] Kataoka H, Yang K, Rock KL. 2015 The xanthine oxidase inhibitor Febuxostat reduces tissue uric acid content and inhibits injury-induced inflammation in the liver and lung. Eur. J. Pharmacol. **746**, 174-179. (10.1016/j.ejphar.2014.11.013) 25449036 PMC4281294

[100] Kono H, Chen CJ, Ontiveros F, Rock KL. 2010 Uric acid promotes an acute inflammatory response to sterile cell death in mice. J. Clin. Invest. **120**, 1939-1949. (10.1172/JCI40124) 20501947 PMC2877935

[101] Yu MA, Sanchez-Lozada LG, Johnson RJ, Kang DH. 2010 Oxidative stress with an activation of the renin-angiotensin system in human vascular endothelial cells as a novel mechanism of uric acid-induced endothelial dysfunction. J. Hypertens. **28**, 1234-1242. (10.1097/HJH.0b013e328337da1d) 20486275

[102] Kanellis J et al. 2003 Uric acid stimulates monocyte chemoattractant protein-1 production in vascular smooth muscle cells via mitogen-activated protein kinase and cyclooxygenase-2. Hypertension **41**, 1287-1293. (10.1161/01.HYP.0000072820.07472.3B) 12743010

[103] Kang DH, Park SK, Lee IK, Johnson RJ. 2005 Uric acid-induced C-reactive protein expression: implication on cell proliferation and nitric oxide production of human vascular cells. J. Am. Soc. Nephrol. **16**, 3553-3562. (10.1681/ASN.2005050572) 16251237

[104] Mirtschink P, Krek W. 2016 Hypoxia-driven glycolytic and fructolytic metabolic programs: pivotal to hypertrophic heart disease. Biochim. Biophys. Acta **1863**, 1822-1828. (10.1016/j.bbamcr.2016.02.011) 26896647

[105] Kanbay M, Altintas A, Yavuz F, Copur S, Sanchez-Lozada LG, Lanaspa MA, Johnson RJ. 2023 Responses to hypoxia: how fructose metabolism and hypoxia-inducible factor-1a pathways converge in health and disease. Curr. Nutr. Rep. **12**, 181-190. (10.1007/s13668-023-00452-5) 36708463

[106] Sharma K et al. 2013 Metabolomics reveals signature of mitochondrial dysfunction in diabetic kidney disease. J. Am. Soc. Nephrol. **24**, 1901-1912. (10.1681/ASN.2013020126) 23949796 PMC3810086

[107] Sas KM et al. 2016 Tissue-specific metabolic reprogramming drives nutrient flux in diabetic complications. JCI Insight **1**, e86976. (10.1172/jci.insight.86976) 27699244 PMC5033761

[108] Lanaspa MA et al. 2014 Endogenous fructose production and fructokinase activation mediate renal injury in diabetic nephropathy. J. Am. Soc. Nephrol. **25**, 2526-2538. (10.1681/ASN.2013080901) 24876114 PMC4214522

[109] Nakagawa T, Sanchez-Lozada LG, Andres-Hernando A, Kojima H, Kasahara M, Rodriguez-Iturbe B, Bjornstad P, Lanaspa MA, Johnson RJ. 2021 Endogenous fructose metabolism could explain the Warburg effect and the protection of SGLT2 inhibitors in chronic kidney disease. Front. Immunol. **12**, 694457. (10.3389/fimmu.2021.694457) 34220855 PMC8243983

[110] Marton A, Kaneko T, Kovalik JP, Yasui A, Nishiyama A, Kitada K, Titze J. 2021 Organ protection by SGLT2 inhibitors: role of metabolic energy and water conservation. Nat. Rev. Nephrol. **17**, 65-77. (10.1038/s41581-020-00350-x) 33005037

[111] Nakagawa T, Lanaspa MA, Millan IS, Fini M, Rivard CJ, Sanchez-Lozada LG, Andres-Hernando A, Tolan DR, Johnson RJ. 2020 Fructose contributes to the Warburg effect for cancer growth. Cancer Metab. **8**, 16. (10.1186/s40170-020-00222-9) 32670573 PMC7350662

[112] Fini MA et al. 2021 Brief report: the uricase mutation in humans increases our risk for cancer growth. Cancer Metab. **9**, 32. (10.1186/s40170-021-00268-3) 34526149 PMC8444362

[113] Goncalves MD et al. 2019 High-fructose corn syrup enhances intestinal tumor growth in mice. Science **363**, 1345-1349. (10.1126/science.aat8515) 30898933 PMC6487857

[114] Johnson RJ et al. 2009 Hypothesis: could excessive fructose intake and uric acid cause type 2 diabetes? Endocr. Rev. **30**, 96-116. (10.1210/er.2008-0033) 19151107 PMC2647706

[115] Johnson RJ, Segal MS, Sautin Y, Nakagawa T, Feig DI, Kang DH, Gersch MS, Benner S, Sanchez-Lozada LG. 2007 Potential role of sugar (fructose) in the epidemic of hypertension, obesity and the metabolic syndrome, diabetes, kidney disease, and cardiovascular disease. Am. J. Clin. Nutr. **86**, 899-906. 17921363 10.1093/ajcn/86.4.899

[116] Bray GA, Nielsen SJ, Popkin BM. 2004 Consumption of high-fructose corn syrup in beverages may play a role in the epidemic of obesity. Am. J. Clin. Nutr. **79**, 537-543. (10.1093/ajcn/79.4.537) 15051594

[117] Johnson RJ, Sanchez-Lozada LG, Andrews P, Lanaspa MA. 2017 Perspective: a historical and scientific perspective of sugar and its relation with obesity and diabetes. Adv. Nutr. **8**, 412-422. (10.3945/an.116.014654) 28507007 PMC5421126

[118] Nardocci M, Polsky JY, Moubarac JC. 2021 Consumption of ultra-processed foods is associated with obesity, diabetes and hypertension in Canadian adults. Can. J. Public Health **112**, 421-429. (10.17269/s41997-020-00429-9) 33174128 PMC8076355

[119] Lachapelle MY, Drouin G. 2011 Inactivation dates of the human and guinea pig vitamin C genes. Genetica **139**, 199-207. (10.1007/s10709-010-9537-x) 21140195

[120] Schulte P et al. 2010 The Chicxulub asteroid impact and mass extinction at the Cretaceous-Paleogene boundary. Science **327**, 1214-1218. (10.1126/science.1177265) 20203042

[121] Wong SK, Chin KY, Ima-Nirwana S. 2020 Vitamin C: a review on its role in the management of metabolic syndrome. Int. J. Med. Sci. **17**, 1625-1638. (10.7150/ijms.47103) 32669965 PMC7359392

[122] Vellekoop J, Sluijs A, Smit J, Schouten S, Weijers JW, Sinninghe Damste JS, Brinkhuis H. 2014 Rapid short-term cooling following the Chicxulub impact at the Cretaceous-Paleogene boundary. Proc. Natl Acad. Sci. USA **111**, 7537-7541. (10.1073/pnas.1319253111) 24821785 PMC4040585

[123] Stavric B, Johnson WJ, Clayman S, Gadd RE, Chartrand A. 1976 Effect of fructose administration on serum urate levels in the uricase inhibited rat. Experientia **32**, 373-374. (10.1007/BF01940847) 1253916

[124] Johnson RJ, Sanchez-Lozada LG, Nakagawa T, Rodriguez-Iturbe B, Tolan D, Gaucher EA, Andrews P, Lanaspa MA. 2022 Do thrifty genes exist? Revisiting uricase. Obesity **30**, 1917-1926. (10.1002/oby.23540) 36150210 PMC9512363

[125] Johnson RJ, Andrews P. 2010 Fructose, uricase, and the back-to-Africa hypothesis. Evol. Anthropol. **19**, 250-257. (10.1002/evan.20266)

[126] Tapia E et al. 2013 Synergistic effect of uricase blockade plus physiological amounts of fructose-glucose on glomerular hypertension and oxidative stress in rats. Am. J. Physiol. Renal. Physiol. **304**, F727-F736. (10.1152/ajprenal.00485.2012) 23303409 PMC3602695

[127] Kratzer JT, Lanaspa MA, Murphy MN, Cicerchi C, Graves CL, Tipton PA, Ortlund EA, Johnson RJ, Gaucher EA. 2014 Evolutionary history and metabolic insights of ancient mammalian uricases. Proc. Natl Acad. Sci. USA **111**, 3763-3768. (10.1073/pnas.1320393111) 24550457 PMC3956161

[128] Johnson RJ, Titte S, Cade JR, Rideout BA, Oliver WJ. 2005 Uric acid, evolution and primitive cultures. Semin. Nephrol. **25**, 3-8. (10.1016/j.semnephrol.2004.09.002) 15660328

[129] Nakagawa T et al. 2006 A causal role for uric acid in fructose-induced metabolic syndrome. Am. J. Physiol. Renal. Physiol. **290**, F625-F631. (10.1152/ajprenal.00140.2005) 16234313

[130] Reungjui S, Roncal CA, Mu W, Srinivas TR, Sirivongs D, Johnson RJ, Nakagawa T. 2007 Thiazide diuretics exacerbate fructose-induced metabolic syndrome. J. Am. Soc. Nephrol. **18**, 2724-2731. (10.1681/ASN.2007040416) 17855639

[131] Nakayama T et al. 2010 Dietary fructose causes tubulointerstitial injury in the normal rat kidney. Am. J. Physiol. Renal. Physiol. **298**, F712-F720. (10.1152/ajprenal.00433.2009) 20071464 PMC2838595

[132] Roncal-Jimenez CA et al. 2011 Sucrose induces fatty liver and pancreatic inflammation in male breeder rats independent of excess energy intake. Metabolism **60**, 1259-1270. (10.1016/j.metabol.2011.01.008) 21489572 PMC3137694

[133] Ishimoto T et al. 2013 Serum from minimal change patients in relapse increases CD80 expression in cultured podocytes. Pediatr. Nephrol. **28**, 1803-1812. (10.1007/s00467-013-2498-4) 23689904 PMC3723775

[134] Lanaspa MA et al. 2018 Ketohexokinase C blockade ameliorates fructose-induced metabolic dysfunction in fructose-sensitive mice. J. Clin. Invest. **128**, 2226-2238. (10.1172/JCI94427) 29533924 PMC5983342

[135] Mrosovsky N, Sherry DF. 1980 Animal anorexias. Science **207**, 837-842. (10.1126/science.6928327) 6928327

[136] Florant GL, Healy JE. 2012 The regulation of food intake in mammalian hibernators: a review. J. Comp. Physiol. B **182**, 451-467. (10.1007/s00360-011-0630-y) 22080368

[137] Jansson JO et al. 2018 Body weight homeostat that regulates fat mass independently of leptin in rats and mice. Proc. Natl Acad. Sci. USA **115**, 427-432. (10.1073/pnas.1715687114) 29279372 PMC5777058

[138] Bake T, Peris-Sampedro F, Waczek Z, Ohlsson C, Palsdottir V, Jansson JO, Dickson SL. 2021 The gravitostat protects diet-induced obese rats against fat accumulation and weight gain. J. Neuroendocrinol. **33**, e12997. (10.1111/jne.12997) 34240761

[139] Ohlsson C, Gidestrand E, Bellman J, Larsson C, Palsdottir V, Hagg D, Jansson PA, Jansson JO. 2020 Increased weight loading reduces body weight and body fat in obese subjects: a proof of concept randomized clinical trial. EClinicalMedicine **22**, 100338. (10.1016/j.eclinm.2020.100338) 32510046 PMC7264953

[140] Van Der Klaauw AA et al. 2016 Divergent effects of central melanocortin signalling on fat and sucrose preference in humans. Nat. Commun. **7**, 13055. (10.1038/ncomms13055) 27701398 PMC5059464

[141] Frank CL. 1994 Polyunsaturate content and diet selection in ground squirrels (*Spermophilus lateralis*). Ecology **75**, 458-463. (10.2307/1939549)

[142] Sclafani A, Ackroff K. 2022 Fat preference deficits and experience-induced recovery in global taste-deficient Trpm5 and Calhm1 knockout mice. Physiol. Behav. **246**, 113695. (10.1016/j.physbeh.2022.113695) 34998826 PMC8826513

[143] Li M, Tan HE, Lu Z, Tsang KS, Chung AJ, Zuker CS. 2022 Gut-brain circuits for fat preference. Nature **610**, 722-730. (10.1038/s41586-022-05266-z) 36070796 PMC9605869

[144] Ishimoto T et al. 2013 High-fat and high-sucrose (western) diet induces steatohepatitis that is dependent on fructokinase. Hepatology **58**, 1632-1643. (10.1002/hep.26594) 23813872 PMC3894259

[145] Roncal-Jimenez CA et al. 2016 Aging-associated renal disease in mice is fructokinase dependent. Am. J. Physiol. Renal. Physiol. **311**, F722-F730. (10.1152/ajprenal.00306.2016) 27465991 PMC5142232

[146] Shephard RJ, Hatcher J, Rode A. 1973 On the body composition of the eskimo. Europ. J. Appl. Physiol. **32**, 3-15. (10.1007/BF00422425)

[147] Rumessen JJ, Gudmand-Hoyer E. 1986 Absorption capacity of fructose in healthy adults. Comparison with sucrose and its constituent monosaccharides. Gut **27**, 1161-1168. (10.1136/gut.27.10.1161) 3781328 PMC1433856

[148] Ushijima K, Riby JE, Fujisawa T, Kretchmer N. 1995 Absorption of fructose by isolated small intestine of rats is via a specific saturable carrier in the absence of glucose and by the disaccharidase-related transport system in the presence of glucose. J. Nutr. **125**, 2156-2164. (10.1093/jn/125.8.2156) 7643250

[149] Ludwig DS et al. 2021 The carbohydrate-insulin model: a physiological perspective on the obesity pandemic. Am. J. Clin. Nutr. **114**, 1873-1885. (10.1093/ajcn/nqab270) 34515299 PMC8634575

[150] Raubenheimer D, Simpson SJ. 2019 Protein leverage: theoretical foundations and ten points of clarification. Obesity (Silver Spring) **27**, 1225-1238. (10.1002/oby.22531) 31339001

[151] Hill CM et al. 2019 FGF21 signals protein status to the brain and adaptively regulates food choice and metabolism. Cell Rep. **27**, 2934-2947. (10.1016/j.celrep.2019.05.022) 31167139 PMC6579533

[152] Van Dam RM, Willett WC, Rimm EB, Stampfer MJ, Hu FB. 2002 Dietary fat and meat intake in relation to risk of type 2 diabetes in men. Diabetes Care **25**, 417-424. (10.2337/diacare.25.3.417) 11874924

[153] Schulze MB, Manson JE, Willett WC, Hu FB. 2003 Processed meat intake and incidence of type 2 diabetes in younger and middle-aged women. Diabetologia **46**, 1465-1473. (10.1007/s00125-003-1220-7) 14576980

[154] Song Y, Manson JE, Buring JE, Liu S. 2004 A prospective study of red meat consumption and type 2 diabetes in middle-aged and elderly women: the women's health study. Diabetes Care **27**, 2108-2115. (10.2337/diacare.27.9.2108) 15333470

[155] Fung TT, Schulze M, Manson JE, Willett WC, Hu FB. 2004 Dietary patterns, meat intake, and the risk of type 2 diabetes in women. Arch. Intern. Med. **164**, 2235-2240. (10.1001/archinte.164.20.2235) 15534160

[156] Wang Y, Beydoun MA. 2009 Meat consumption is associated with obesity and central obesity among US adults. Int. J. Obes. **33**, 621-628. (10.1038/ijo.2009.45) PMC2697260 19308071

[157] Lew QJ, Jafar TH, Koh HW, Jin A, Chow KY, Yuan JM, Koh WP. 2017 Red meat intake and risk of ESRD. J. Am. Soc. Nephrol. **28**, 304-312. (10.1681/ASN.2016030248) 27416946 PMC5198288

[158] Kaneko K, Kudo Y, Yamanobe T, Mawatari K, Yasuda M, Nakagomi K, Fujimori S. 2008 Purine contents of soybean-derived foods and selected Japanese vegetables and mushrooms. Nucleosides Nucleotides Nucleic Acids **27**, 628-630. (10.1080/15257770802138681) 18600517

[159] Feigelson M, Feigelson P. 1966 Relationships between hepatic enzyme induction, glutamate formation, and purine nucleotide biosynthesis in glucocorticoid action. J. Biol. Chem. **241**, 5819-5826. (10.1016/S0021-9258(18)96346-5) 4380931

[160] Shimizu T, Hiroata R, Nomura Y, Aibara K, Miyaki K. 1971 Nephrotoxic effect of MSG (monosodium glutamate) in the chicken. Jpn. J. Med. Sci. Biol. **24**, 271-279. (10.7883/yoken1952.24.271) 5316781

[161] Flynn TJ et al. 2015 Positive association of tomato consumption with serum urate: support for tomato consumption as an anecdotal trigger of gout flares. BMC Musculoskelet. Disord. **16**, 196. (10.1186/s12891-015-0661-8) 26286027 PMC4541734

[162] Gutman AB, Yu TF. 1973 Hyperglutamatemia in primary gout. Am. J. Med. **54**, 713-724. (10.1016/0002-9343(73)90057-0) 4705416

[163] Choi HK, Atkinson K, Karlson EW, Willett W, Curhan G. 2004 Purine-rich foods, dairy and protein intake, and the risk of gout in men. N. Engl. J. Med. **350**, 1093-1103. (10.1056/NEJMoa035700) 15014182

[164] Johnson RJ et al. 2013 Umami: the taste that drives purine intake. J. Rheumatol. **40**, 1794-1796. (10.3899/jrheum.130531) 24187156

[165] Minami S, Sato M, Shiraiwa Y, Iwamoto K. 2011 Molecular characterization of adenosine 5'-monophosphate deaminase–the key enzyme responsible for the umami taste of nori (*Porphyra yezoensis Ueda,* Rhodophyta). Mar. Biotechnol. **13**, 1140-1147. (10.1007/s10126-011-9377-4) 21519809

[166] Lanaspa MA et al. 2012 Uric acid stimulates fructokinase and accelerates fructose metabolism in the development of fatty liver. PLoS ONE **7**, e47948. (10.1371/journal.pone.0047948) 23112875 PMC3480441

[167] Maier S, Wieland A, Cree-Green M, Nadeau K, Sullivan S, Lanaspa MA, Johnson RJ, Jensen T. 2021 Lean NAFLD: an underrecognized and challenging disorder in medicine. Rev. Endocr. Metab. Disord. **22**, 351-366. (10.1007/s11154-020-09621-1) 33389543 PMC8893229

[168] Wolf JP, Nguyen NU, Dumoulin G, Berthelay S. 1992 Influence of hypertonic monosaccharide infusions on the release of plasma arginine vasopressin in normal humans. Horm. Metab. Res. **24**, 379-383. (10.1055/s-2007-1003340) 1526626

[169] Song Z et al. 2017 Role of fructose and fructokinase in acute dehydration-induced vasopressin gene expression and secretion in mice. J. Neurophysiol. **117**, 646-654. (10.1152/jn.00781.2016) 27852737 PMC5288484

[170] Ortiz RM. 2001 Osmoregulation in marine mammals. J. Exp. Biol. **204**, 1831-1844. (10.1242/jeb.204.11.1831) 11441026

[171] Johnson RJ, Stenvinkel P, Jensen T, Lanaspa MA, Roncal C, Song Z, Bankir L, Sanchez-Lozada LG. 2016 Metabolic and kidney diseases in the setting of climate change, water shortage, and survival factors. J. Am. Soc. Nephrol. **27**, 2247-2256. (10.1681/ASN.2015121314) 27283495 PMC4978060

[172] Stookey JD, Kavouras S, Suh H, Lang F. 2020 Underhydration is associated with obesity, chronic diseases, and death within 3, to 6 years in the U.S. population aged 51–70 years. Nutrients **12**, 905. (10.3390/nu12040905) 32224908 PMC7230456

[173] Stookey JD, Barclay D, Arieff A, Popkin BM. 2007 The altered fluid distribution in obesity may reflect plasma hypertonicity. Eur. J. Clin. Nutr. **61**, 190-199. (10.1038/sj.ejcn.1602521) 17021599

[174] Enhorning S, Struck J, Wirfalt E, Hedblad B, Morgenthaler NG, Melander O. 2011 Plasma copeptin, a unifying factor behind the metabolic syndrome. J. Clin. Endocrinol. Metab. **96**, E1065-E1072. (10.1210/jc.2010-2981) 21490073

[175] Li Q et al. 2021 Obesity and hyperinsulinemia drive adipocytes to activate a cell cycle program and senesce. Nat. Med. **27**, 1941-1953. (10.1038/s41591-021-01501-8) 34608330

[176] Watanabe S, Kang DH, Feng L, Nakagawa T, Kanellis J, Lan H, Mazzali M, Johnson RJ. 2002 Uric acid, hominoid evolution, and the pathogenesis of salt-sensitivity. Hypertension **40**, 355-360. (10.1161/01.HYP.0000028589.66335.AA) 12215479

[177] Rodriguez-Iturbe B, Pons H, Johnson RJ. 2017 Role of the immune system in hypertension. Physiol. Rev. **97**, 1127-1164. (10.1152/physrev.00031.2016) 28566539 PMC6151499

[178] Abdelmalek MF, Suzuki A, Guy C, Unalp-Arida A, Colvin R, Johnson RJ, Diehl AM. 2010 Increased fructose consumption is associated with fibrosis severity in patients with nonalcoholic fatty liver disease. Hepatology **51**, 1961-1971. (10.1002/hep.23535) 20301112 PMC2922495

[179] Harchaoui KE, Visser ME, Kastelein JJ, Stroes ES, Dallinga-Thie GM. 2009 Triglycerides and cardiovascular risk. Curr. Cardiol. Rev. **5**, 216-222. (10.2174/157340309788970315) 20676280 PMC2822144

[180] Sanchez-Lozada LG, Tapia E, Johnson RJ, Rodriguez-Iturbe B, Herrera-Acosta J. 2003 Glomerular hemodynamic changes associated with arteriolar lesions and tubulointerstitial inflammation. Kidney Int. Suppl. **64**, S9-14. (10.1046/j.1523-1755.64.s86.3.x) 12969121

[181] Klauser AS, Halpern EJ, Strobl S, Gruber J, Feuchtner G, Bellmann-Weiler R, Weiss G, Stofferin H, Jaschke W. 2019 Dual-energy computed tomography detection of cardiovascular monosodium urate deposits in patients with gout. JAMA Cardiol. **4**, 1019-1028. (10.1001/jamacardio.2019.3201) 31509156 PMC6739730

[182] Ejaz AA, Antenor JA, Kumar V, Roncal C, Garcia GE, Andres-Hernando A, Lanaspa MA, Johnson RJ. 2022 Uric acid: a friend in the past, a foe in the present. Integr. Med. Nephrol. Androl. **9**, 8. (10.4103/2773-0387.348714)

[183] Mirtschink P, Jang C, Arany Z, Krek W. 2018 Fructose metabolism, cardiometabolic risk, and the epidemic of coronary artery disease. Eur. Heart J. **39**, 2497-2505. (10.1093/eurheartj/ehx518) 29020416 PMC6037111

[184] Hahn K, Rodriguez-Iturbe B, Winterberg B, Sánchez-Lozada L, Kanbay M, Lanaspa M, Johnson R. 2022 Primary aldosteronism: a consequence of sugar and western diet? Med. Hypotheses **160**, 110796. (10.1016/j.mehy.2022.110796)

[185] Ridker PM. 2020 Targeting interleukin-1 and interleukin-6: the time has come to aggressively address residual inflammatory risk. J. Am. Coll. Cardiol. **76**, 1774-1776. (10.1016/j.jacc.2020.08.052) 33032739

[186] Nardi V et al. 2022 Uric acid expression in carotid atherosclerotic plaque and serum uric acid are associated with cerebrovascular events. Hypertension **79**, 1814-1823. (10.1161/HYPERTENSIONAHA.122.19247) 35656807

[187] Kleber ME, Delgado G, Grammer TB, Silbernagel G, Huang J, Kramer BK, Ritz E, Marz W. 2015 Uric acid and cardiovascular events: a Mendelian randomization study. J. Am. Soc. Nephrol. **26**, 2831-2838. (10.1681/ASN.2014070660) 25788527 PMC4625666

[188] Kuo CF, See LC, Yu KH, Chou IJ, Chiou MJ, Luo SF. 2012 Significance of serum uric acid levels on the risk of all-cause and cardiovascular mortality. Rheumatology **52**, 127-134. (10.1093/rheumatology/kes223) 22923756

[189] Stenvinkel P, Meyer CJ, Block GA, Chertow GM, Shiels PG. 2020 Understanding the role of the cytoprotective transcription factor nuclear factor erythroid 2-related factor 2—lessons from evolution, the animal kingdom and rare progeroid syndromes. Nephrol. Dial. Transplant. **35**, 2036-2045. (10.1093/ndt/gfz120) 31302696 PMC7716811

[190] Amani S, Fatima S. 2020 Glycation with fructose: the bitter side of nature's own sweetener. Curr. Diabetes Rev. **16**, 962-970. (10.2174/1389450121666200204115751) 32013850

[191] Levi B, Werman MJ. 1998 Long-term fructose consumption accelerates glycation and several age-related variables in male rats. J. Nutr. **128**, 1442-1449. (10.1093/jn/128.9.1442) 9732303

[192] Hernandez-Rios R et al. 2013 Low fructose and low salt diets increase mitochondrial DNA in white blood cells of overweight subjects. Exp. Clin. Endocrinol. Diabetes **121**, 535-538. (10.1055/s-0033-1349144) 23934680

[193] Johnson RJ, Andrews P. 2015 The fat gene: a genetic mutation in prehistoric apes may underlie today's pandemic of obesity and diabetes. Sci. Am. **313**, 64-69. (10.1038/scientificamerican1015-64)

[194] Johnson RJ, Lanaspa MA, Fox JW. 2021 Upper Paleolithic figurines showing women with obesity may represent survival symbols of climatic change. Obesity **29**, 11-15. (10.1002/oby.23028) 33258218 PMC7902358

[195] Evershed RP et al. 2022 Dairying, diseases and the evolution of lactase persistence in Europe. Nature **608**, 336-345. (10.1038/s41586-022-05010-7) 35896751 PMC7615474

[196] Speakman JR. 2018 The evolution of body fatness: trading off disease and predation risk. J. Exp. Biol. **221**, jeb.167254. (10.1242/jeb.167254) 29514887

[197] Diamond J. 2003 The double puzzle of diabetes. Nature **423**, 599-602. (10.1038/423599a) 12789325

[198] Vercammen KA, Moran AJ, Soto MJ, Kennedy-Shaffer L, Bleich SN. 2020 Decreasing trends in heavy sugar-sweetened beverage consumption in the United States, 2003 to 2016. J. Acad. Nutr. Diet **120**, 1974-1985. (10.1016/j.jand.2020.07.012) 32981886

[199] Ng SW, Slining MM, Popkin BM. 2012 Use of caloric and noncaloric sweeteners in US consumer packaged foods, 2005–2009. J. Acad. Nutr. Diet **112**, 1828-1834. (10.1016/j.jand.2012.07.009) 23102182 PMC3490437

[200] Monteiro CA, Moubarac JC, Cannon G, Ng SW, Popkin B. 2013 Ultra-processed products are becoming dominant in the global food system. Obes. Rev. **14**, 21-28. (10.1111/obr.12107) 24102801

[201] Luukkonen PK et al. 2018 Saturated fat is more metabolically harmful for the human liver than unsaturated fat or simple sugars. Diabetes Care **41**, 1732-1739. (10.2337/dc18-0071) 29844096 PMC7082640

[202] Guasch-Ferre M et al. 2017 Total and subtypes of dietary fat intake and risk of type 2 diabetes mellitus in the Prevencion con Dieta Mediterranea (PREDIMED) study. Am. J. Clin. Nutr. **105**, 723-735. (10.3945/ajcn.116.142034) 28202478

[203] Lee JH, Duster M, Roberts T, Devinsky O. 2021 United States Dietary Trends Since 1800: lack of association between saturated fatty acid consumption and non-communicable diseases. Front. Nutr. **8**, 748847. (10.3389/fnut.2021.748847) 35118102 PMC8805510

[204] Jensen T et al. 2018 Fructose and sugar: a major mediator of non-alcoholic fatty liver disease. J. Hepatol. **68**, 1063-1075. (10.1016/j.jhep.2018.01.019) 29408694 PMC5893377

[205] Goldberg EL et al. 2017 Beta-hydroxybutyrate deactivates neutrophil nlrp3 inflammasome to relieve gout flares. Cell Rep. **18**, 2077-2087. (10.1016/j.celrep.2017.02.004) 28249154 PMC5527297

[206] Andrews C, Zuidersma E, Verhulst S, Nettle D, Bateson M. 2021 Exposure to food insecurity increases energy storage and reduces somatic maintenance in European starlings (*Sturnus vulgaris*). R. Soc. Open Sci. **8**, 211099. (10.1098/rsos.211099) 34540262 PMC8441118

[207] Grabek KR, Cooke TF, Epperson LE, Spees KK, Cabral GF, Sutton SC, Merriman DK, Martin SL, Bustamante CD. 2019 Genetic variation drives seasonal onset of hibernation in the 13-lined ground squirrel. Commun. Biol. **2**, 478. (10.1038/s42003-019-0719-5) 31886416 PMC6925185

[208] Levy O, Dayan T, Porter WP, Kronfeld-Schor N. 2016 Foraging activity pattern is shaped by water loss rates in a diurnal desert rodent. Am. Nat. **188**, 205-218. (10.1086/687246) 27420785

[209] Feig DI, Soletsky B, Johnson RJ. 2008 Effect of allopurinol on blood pressure of adolescents with newly diagnosed essential hypertension: a randomized trial. J. Am. Med. Assoc. **300**, 924-932. (10.1001/jama.300.8.924) PMC2684336 18728266

